# ENO1-mediated deoxycytidine synthesis and gemcitabine resistance by stabilizing RRM2 in pancreatic cancer

**DOI:** 10.1038/s41419-025-08061-6

**Published:** 2025-12-27

**Authors:** Yongning Li, Hao Wang, Liwen Chen, Yanyu Gong, Dijie Zheng, Futang Li, Changhao Wu, Zhiwei He, Chao Yu

**Affiliations:** 1 Department of Hepatobiliary Surgery, The Affiliated Hospital of Guizhou Medical University, Guizhou Medical University, Guiyang, China; 2 School of Clinical Medicine, Guizhou Medical University, Guiyang, China; 3 Guizhou Provincial Institute of Hepatobiliary, Pancreatic and Splenic Diseases, Guiyang, China; 4 Key Laboratory of Liver, Gallbladder, Pancreas and Spleen of Guizhou Medical University, Guiyang, China; 5 Guizhou Provincial Clinical Medical Research Center of Hepatobiliary Surgery, Guiyang, Guizhou China; 6 Key Laboratory of Hepatobiliary and Pancreatic Diseases Treatment and Bioinformatics Research, Guizhou Medical University, Guiyang, China

**Keywords:** Cancer metabolism, Cancer therapeutic resistance

## Abstract

Pancreatic ductal adenocarcinoma is a highly malignant solid tumor of the digestive tract, and chemoresistance to gemcitabine is an important cause of shortened survival time in patients. Upregulation of deoxypyrimidine synthesis is one of the important reasons for pancreatic cancer cells to be resistant to gemcitabine, however, the specific mechanism leading to increased deoxypyrimidine synthesis in pancreatic cancer cells is still unclear. Ribonucleotide reductase M2 subunit (RRM2) is overexpressed through unclear mechanisms in many types of human cancer significantly affects sensitivity to various chemotherapy treatments. Here, we found that high expression of enolase-1 (ENO1) is closely related to gemcitabine resistance in pancreatic cancer patients. Cellular experiments and in vivo experiments confirmed that ENO1 increases the resistance of pancreatic cancer to gemcitabine without relying on its glycolytic enzyme activity. Mechanistically, ENO1 competitively binds to RRM2 with ubiquitin E3 ligase STUB1, thereby weakening the ubiquitination and degradation of RRM2 by STUB1. This ENO1-mediated aggregation of RRM2 protein increases the synthesis of dNTPs in pancreatic cancer cells, enhancing the resistance of pancreatic cancer to gemcitabine. Our study reveals a role of ENO1 in pancreatic cancer via RRM2-STUB1 axis and provides a scientific basis for the development of new therapeutic strategies targeting ENO1.

## Introduction

Pancreatic cancer, over 90% of which is pancreatic ductal adenocarcinoma (PDAC), is a highly aggressive gastrointestinal tumor [1]. Due to its insidious onset, lack of effective early screening markers, fewer than 20% of patients are eligible for surgical resection [2, 3]. Over the past few decades, significant advancements in cancer treatment have led to the development of targeted and immunotherapeutic drugs that have greatly improved the prognosis of various malignancies. However, these drugs do not yield satisfactory outcomes in patients with pancreatic cancer [4–6]. Currently, gemcitabine remains the first-line treatment for pancreatic cancer [7]. However, resistance to gemcitabine results in poor therapeutic responses in over 90% of patients [8–10]. Therefore, elucidating the molecular basis of gemcitabine resistance in pancreatic cancer is crucial for precisely selecting patients who will benefit from gemcitabine treatment, developing new drugs to enhance its efficacy, and improving the prognosis of pancreatic cancer.

Gemcitabine is a deoxycytidine nucleoside analog, upon cellular uptake, is converted into a deoxynucleoside diphosphate(dCDP) analog [11], which inhibits ribonucleotide reductase, an enzyme required for the production of deoxyribonucleotide triphosphates(dCTP), thereby reducing intracellular nucleotides [12]. Once gemcitabine is phosphorylated to its triphosphate form, it competes with dCTP and incorporates into nascent DNA, inducing apoptosis [13]. Multiple mechanisms contribute to its resistance, including aberrant metabolism [14, 15] and transport of gemcitabine [16, 17], and enhanced DNA repair mechanisms [18]. Recent studies have suggested that abnormal pyrimidine synthesis in pancreatic cancer cells leads to increased intracellular dCTP levels, competitively inhibiting the activity of gemcitabine and enhancing its resistance [19–21]. The ribonucleotide reductase M2 subunit (RRM2), a key rate-limiting enzyme in dCTP synthesis, is overexpressed in many types of human cancers, including pancreatic cancer [22–24], and is associated with chemoresistance [25, 26]. However, specific regulatory mechanisms underlying its expression remain unclear.

Enolase-1 (ENO1) catalyzes the interconversion of 2-phosphoglycerate and phosphoenolpyruvate during glycolysis and is widely distributed in most tissues [27]. Beyond its classical glycolytic enzyme activity, ENO1 has been shown to have multiple functions, such as acting as a strong plasminogen receptor to promote extracellular matrix degradation [28], regulating RNA stability and translation as an RNA-binding protein [29], and serving as a chaperone for heat shock proteins, as well as cytoskeletal [28]. ENO1 is overexpressed in various cancers and is closely associated with patient prognosis [30]. Our previous findings revealed that ENO1 promotes the progression of pancreatic cancer by activating the PIK-AKT pathway [31], but its specific roles and mechanisms in pancreatic cancer remain unclear.

This study aimed to investigate the role of ENO1 in mediating gemcitabine resistance in pancreatic cancer. Specifically, we examined the molecular interactions between ENO1, RRM2, and the E3 ligase STUB1 to determine how ENO1 influences deoxycytidine synthesis and chemoresistance. Our study seeks to identify a new role of ENO1 in pancreatic cancer chemoresistance and provide a scientific basis for the development of new therapeutic strategies targeting ENO1.

## Result

### High ENO1 expression in pancreatic cancer correlates with gemcitabine resistance

**Fig. 1:**
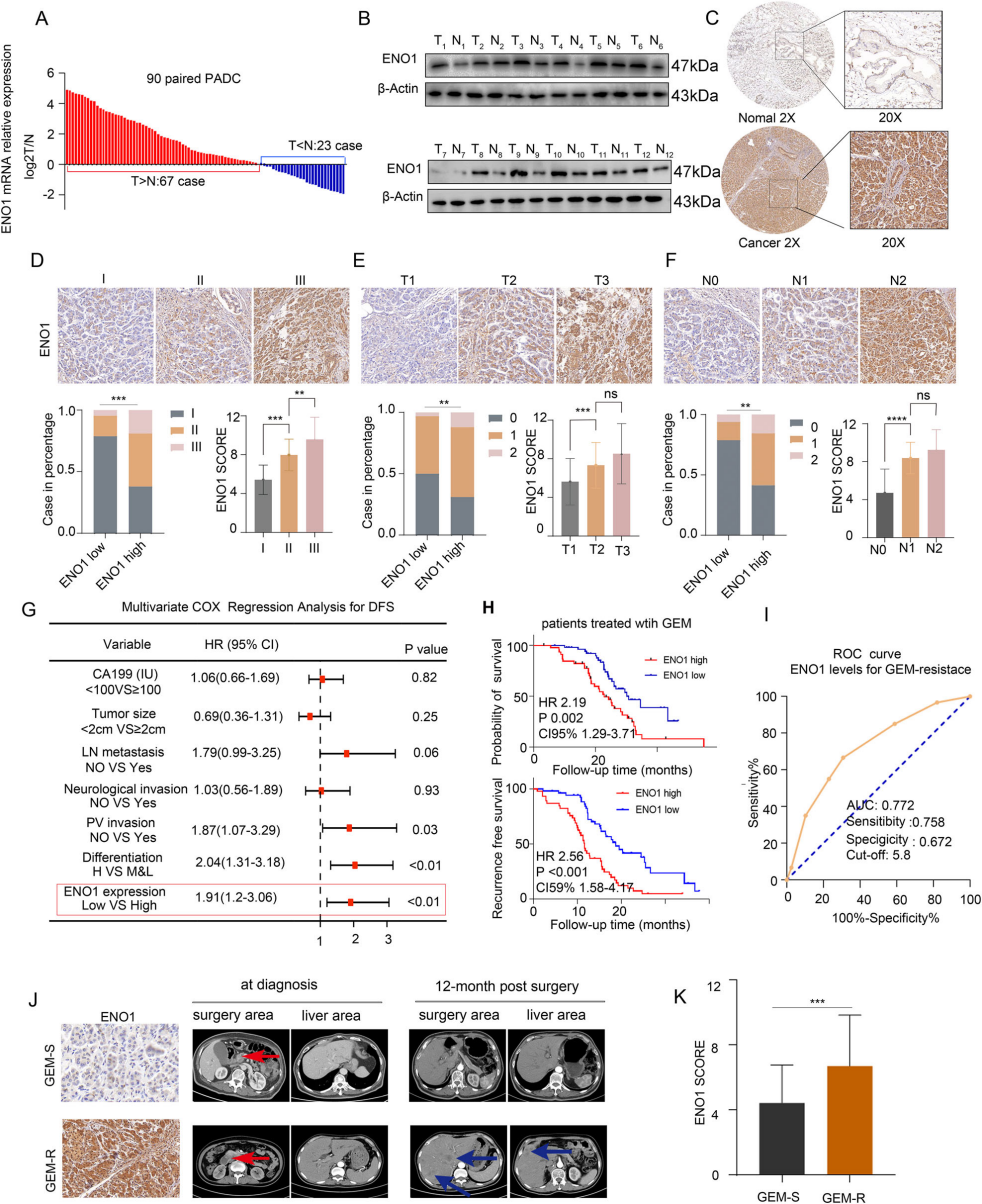
High ENO1 expression in pancreatic cancer correlates with gemcitabine resistance. **A** qPCR analyzed ENO1 mRNA levels in 90 pancreatic cancer and adjacent tissues. **B** Western blotting was employed to assess the protein expression levels of ENO1 in 12 pairs of fresh pancreatic cancer and adjacent tissues. **C** Representative immunohistochemical images of ENO1 expression in paired pancreatic cancer tissue samples. **D–F** Representative IHC staining images of pancreatic cancer tissues based on AJCC staging, T grade, and N stage, along with ENO1 staining scores and distribution of high and low expression in each group.The data were analyzed using analysis of variance (ANOVA). **G** Forest plot of multivariate COX regression analysis for recurrence-free survival in pancreatic cancer patients. **H** Impact of ENO1 expression levels on overall survival (OS) and recurrence-free survival (RFS) in pancreatic cancer patients who received gemcitabine treatment. **I** ROC curve showing the diagnostic value of ENO1 expression levels for gemcitabine resistance in 100 pancreatic cancer patients. **J** ENO1 IHC staining and corresponding CT images in gemcitabine-sensitive and gemcitabine-resistant patients. Red arrows indicate primary tumors; blue arrows indicate liver metastases. **K** Statistical graphs of ENO1 IHC in gemcitabine-sensitive and gemcitabine-resistant patients analyzed using a two-tailed Student’s t-test.

To investigate the expression pattern of ENO1 in pancreatic cancer, we analyzed data from the TCGA database and found that ENO1 expression was significantly higher in pancreatic cancer tissue compared to normal pancreatic tissue (Fig. S1A). Additionally, we assessed ENO1 expression in 90 pairs of matched pancreatic cancer and adjacent non-cancerous tissues using quantitative polymerase chain reaction (qPCR) and found higher ENO1 levels in most cancer tissues (Fig. 1A). Western blot (WB) and immunohistochemistry (IHC) analysis further substantiated these findings (Fig. 1B and C). To further clarify the clinical significance of ENO1 expression in pancreatic cancer, we retrospectively collected clinical and follow-up data from patients who underwent curative surgery and pathologically diagnosed with PDAC at our hospital. The patient recruitment process is illustrated in Fig. S1D. Patients were divided into high- and low-expression groups based on immunohistochemical scores. Table S1 summarizes the clinical baseline characteristics of the two groups. Compared to the low-expression group, the high ENO1 expression group exhibited higher T stages, higher AJCC stages, more lymph node metastases. Representative IHC images of ENO1 expression based on AJCC stage, T stage, and lymph node metastasis are shown in Fig. 1D–F. Analysis of patient follow-up data revealed that high ENO1 expression significantly reduced recurrence-free survival (RFS) and overall survival (OS) (Fig. S1 E, F). Similarly, in the TCGA database, the expression of ENO1 is also associated with OS and RFS(Fig. S1B, C). Further, Cox multivariate regression analysis indicated that ENO1 expression was an independent risk factor for RFS (Fig. 1G) but not for OS (Table S2). Given that our study included patients with surgically resected pancreatic cancer who mostly received gemcitabine(GEM)-based adjuvant chemotherapy, subgroup analysis indicated that ENO1 expression was significantly associated with OS and RFS in gemcitabine-treated patients (Fig. 1H). We hypothesized that ENO1 expression is related to gemcitabine resistance. Patients who received gemcitabine adjuvant chemotherapy postoperatively and experienced recurrence within 1 year were defined as gemcitabine resistant. Additionally, multivariate logistic regression analysis indicated that high ENO1 expression was an independent risk factor for gemcitabine resistance (Table S3). Furthermore, receiver operating characteristic (ROC) curve demonstrated that ENO1 levels have a significant diagnostic value for gemcitabine resistance, with an AUC of 0.772 (Fig. 1I). In the gemcitabine-resistant group, the expression level of ENO1 is also significantly increased.(Fig. 1J, K). Overall, high ENO1 expression promotes gemcitabine resistance in patients with pancreatic cancer, thereby affecting their prognosis.

### ENO1 mediates gemcitabine resistance in pancreatic cancer independently of enzymatic activity

**Fig. 2:**
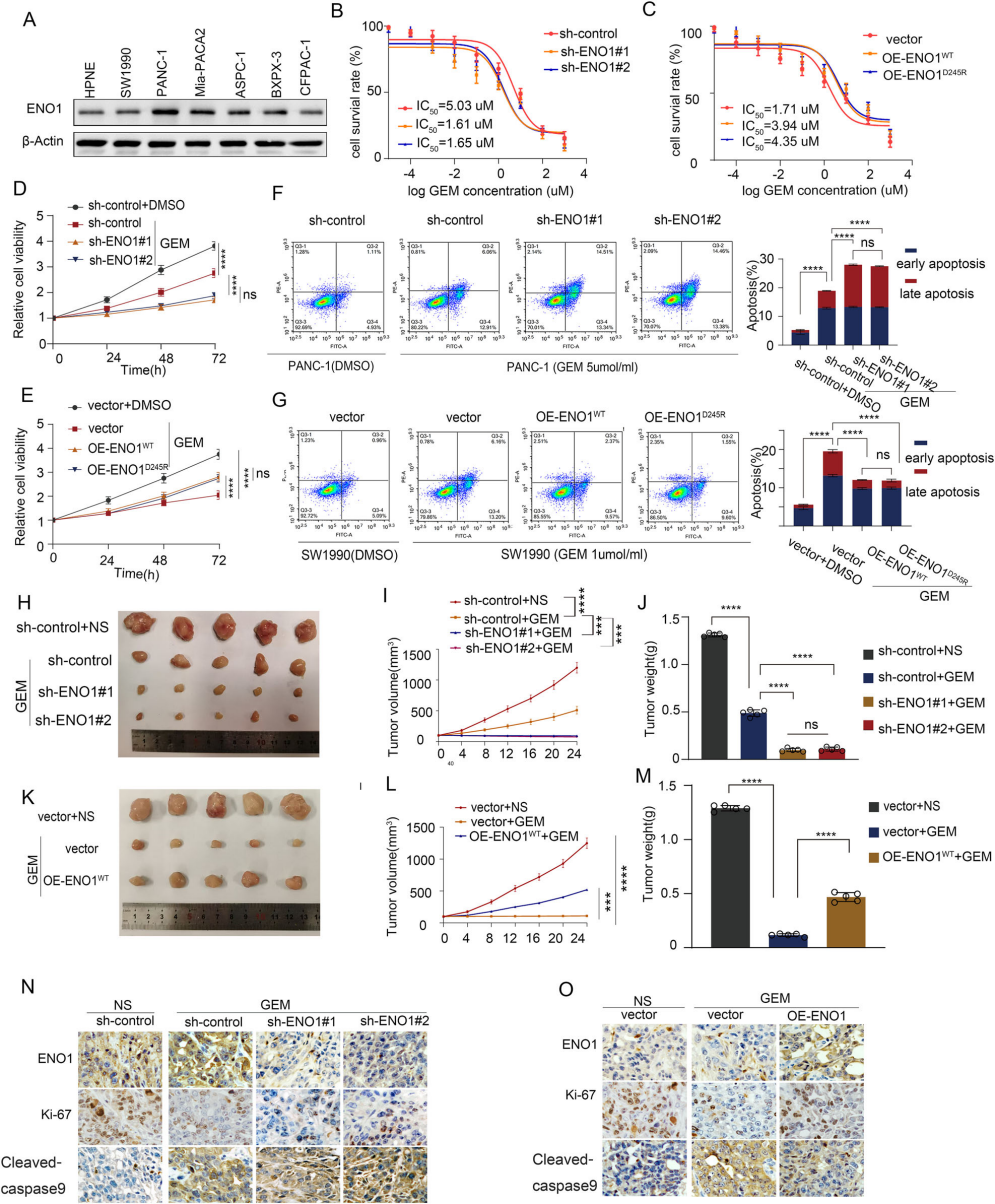
ENO1 mediates gemcitabine resistance in pancreatic cancer independently of enzymatic activity. **A** WB assessed ENO1 expression in pancreatic epithelial and cancer cells. **B**, **C** Impact of ENO1 knockdown in PANC-1 or overexpression of ENO1^WT^/ENO1^D245R^ in SW1990 on sensitivity to GEM. **D**, **E** CCK-8 evaluated proliferation of ENO1 knockdown or overexpressing cell lines post-DMSO or GEM treatment (5 μmol/L GEM for PANC-1, 2 μmol/L for SW1990). The data were analyzed using analysis of variance (ANOVA) **F**, **G** Apoptosis rates and statistics for ENO1 knockdown or overexpressing cell lines post-DMSO or GEM treatment.The data were analyzed using analysis of variance (ANOVA) **H**, **K** PANC-1 with ENO1 knockdown and its control, and SW1990 with ENO1 overexpression and its control were implanted into nude mice. The resulting tumor images (**H**, **K**) and corresponding tumor growth curves (**I**, **L**), as well as tumor weight statistics (**J**, **M**), were obtained following the experimental procedures. The data were analyzed using analysis of variance (ANOVA) **N**, **O** IHC detected ENO1, KI-67, and Caspase3 in respective groups. All in vitro experiments were carried out in three replicates.

To determine whether ENO1 can promote gemcitabine resistance in pancreatic cancer cells, we assessed ENO1 expression in multiple pancreatic cancer cell lines using qPCR and WB, ENO1 was highly expressed in various pancreatic cancer cell lines compared to normal pancreatic epithelial cells. (Figs. S2A and 2A). Next, we knocked down ENO1 in PANC-1 cells and overexpressed ENO1^WT^ in SW1990 cells. Since ENO1 is involved in glycolysis through its enzymatic activity, we aimed to determine whether this enzymatic activity affects the resistance of pancreatic cancer cells to gemcitabine. We also overexpressed the enzymatically inactive mutant ENO1 (ENO1^D245R^) in SW1990 cells and verified its knockdown or overexpression efficiency using qPCR and WB (Fig. S2B, C). After 72 h of gemcitabine treatment, the half-maximal inhibitory concentration (IC50) of gemcitabine in ENO1-knockdown cells was significantly reduced, and increased in cells overexpressing ENO1 (Fig. 2B, C). Intrestingly,the IC50 was also significantly elevated in cells overexpressing ENO1^D245R^ (Fig. 2C). Exposure of pancreatic cancer cells to gemcitabine and subsequent assessment of proliferation using EdU (Fig. S2 D) and CCK8 (Fig. 2D, E) assays revealed that ENO1 knockdown significantly suppressed cell proliferation, while overexpression enhanced the proliferative capacity in the presence of gemcitabine. Similarly, ENO1 knockdown impaired the clonogenic ability of PDAC cell in gemcitabine, whereas overexpression of ENO1^WT^ and ENO1D^245R^ significantly promoted clonogenic formation (Fig. S2E). Given that the antitumor activity of gemcitabine primarily relies on its inhibition of DNA synthesis, which leads to cell cycle arrest, and apoptosis [11], we further investigated the impact of ENO1 on cell cycle progression and apoptosis in gemcitabine-treated cells. The results showed that, compared to the control group, ENO1 knockdown led cell arrest in G1 and S phases (Fig. S2F) and an increase in apoptotic cells after gemcitabine treatment (Fig. 2F, G). In contrast, overexpression of ENO1^WT^ and ENO1D^245R^ had the opposite effect. In summary, ENO1 can promote gemcitabine resistance in pancreatic cancer cells independent of its enzymatic activity.

We established a nude mouse subcutaneous tumor model to further evaluate the effect of ENO1 on gemcitabine resistance in pancreatic cancer cells in vivo. ENO1-knockdown PANC-1 cells, ENO1-overexpressing SW1990 cells, and their respective controls were implanted into nude mice. Consistent with our in vitro findings, ENO1 knockdown significantly inhibited tumor growth (Fig. 2H–J), while overexpression promoted tumor progression (Fig. 2K–M) in the gemcitabine-treated group compared to the control group. IHC analysis of tumor samples for KI-67 and cleaved caspase-9 revealed that ENO1 knockdown significantly reduced cell proliferation and increased apoptosis (Fig. 2N), whereas ENO1 overexpression had the opposite effect (Fig. 2O). To summarize, ENO1 enhances gemcitabine resistance in pancreatic cancer cells in vivo.

### ENO1 binds to RRM2 to enhance the stability of the RRM2 protein

**Fig. 3:**
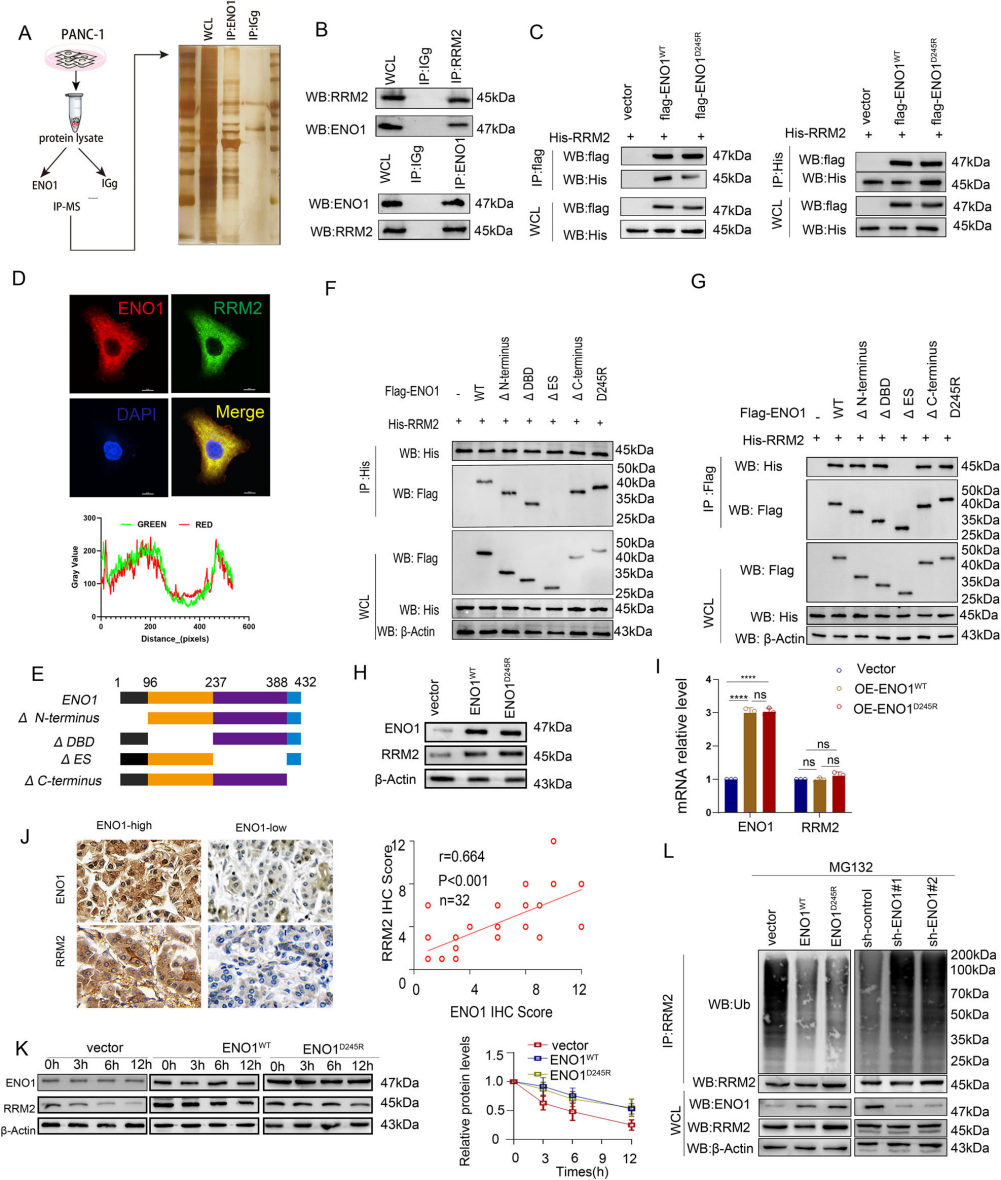
ENO1 binds to RRM2 to enhance the stability of the RRM2 protein. **A** Schematic and silver staining images of IP-MS to screen ENO1-binding proteins, with IgG precipitation as a negative control. **B** Endogenous ENO1 or RRM2 precipitation in PANC-1 cell lysates using specific antibodies, followed by WB detection, with IgG as a negative control. **C** Co-IP assay in HEK293T cells transfected with flag-ENO1^WT^ or ENO1^D245R^ and Myc-RRM2 plasmids. Flag-tag and His antibodies were used for protein precipitation and WB detection. **D** Confocal images showing co-localization of ENO1 (red) and RRM2 (green) in PANC-1 cells, with DAPI staining for nuclei (blue). **E** Schematic diagram of full-length ENO1 and various truncation mutants. **F**, **G**. Co-expression of flag-ENO1 or indicated truncation mutants with His-RRM2 in HEK293T cells. Flag-tag and His antibodies were used for protein precipitation and WB detection. Overexpression of ENO1^WT^ or ENO1^D245R^ in SW1990 cells, with WB (**H**) and qPCR (**I**) to detect protein and mRNA levels of ENO1 and RRM2. Data are analyzed using a two-tailed Student’s t-test. **J** IHC of ENO1 and RRM2 in pancreatic cancer tissues and their correlation. Statistical test: Spearman’s rank correlation. **K** CHX chase analysis of RRM2 in SW1990 cells overexpressing ENO1^WT^ or ENO1^D245R^. Cells expressing an empty vector served as a negative control. Each data point represents the average intensity of protein blots from three replicate experiments at each time point. **L** PANC-1 cells transfected with the indicated sh-RNA (left panel), and SW1990 cells transfected with ENO1^WT^ or ENO1^D245R^ plasmids (right panel). Cell lysates were subjected to IP with an RRM2 antibody, followed by WB detection. Cells were treated with 20 μM MG132 for 8 h before harvest.All in vitro experiments were carried out in three replicates.

To clarify the specific mechanisms by which ENO1 promotes gemcitabine resistance in pancreatic cancer cells.we hypothesized that ENO1 may affect certain proteins to enhance pancreatic cancer resistance to gemcitabine, given that its role in pancreatic cancer is independent of its enzymatic activity. Therefore, we performed immunoprecipitation (IP) on PANC-1 whole-cell lysates analyzed the samples using IP-mass spectrometry (IP-MS) (Fig. 3A). Functional enrichment analysis revealed pyrimidine metabolism as the most prominent pathway (Fig. S3A). Among the proteins identified, RRM2, had the highest peptide count (Fig. S3B). We confirmed the interaction between ENO1 and RRM2 in pancreatic cancer cells using co-immunoprecipitation (co-IP) (Fig. 3B) and in 293 T cells overexpressing HA-RRM2, flag-ENO1^WT^ or flag-ENO1D^245R^ (Fig. 3C). Immunofluorescence co-localization analysis also showed that ENO1 and RRM2 were primarily localized in the cytoplasm (Fig. 3D). To identify the ENO1 region responsible for RRM2 binding, we constructed flag-tagged deletion mutants of ENO1 (Fig. 3E) and performed co-IP assays. The results indicated that the ES domain of ENO1 is the main segment responsible for binding to RRM2 (Fig. 3F, G). Overall, these findings confirm that RRM2 interacts with ENO1.

We aim to investigate the possible implications of the binding between ENO1 and RRM2.We further explored the impact of ENO1 on RRM2 expression. ENO1 knockdown reduced RRM2 protein levels (Fig. S3C), while ENO1 overexpression increased them (Fig. 3H) without affecting RRM2 mRNA levels (Figs. 3I and S3C), suggesting that ENO1 regulates RRM2 expression at the post-transcriptional level. In pancreatic cancer tissues, we detected a moderate positive correlation between ENO1 and RRM2 expression using WB (Fig. S3D) and IHC (Fig. 3J). We hypothesized that ENO1 may affect RRM2 expression at the post-translational level by influencing RRM2 stability. Cyclohexamide (CHX) pulse analysis revealed that overexpression of ENO1^WT^ or ENO1^D245R^ significantly extended the half-life of RRM2 (Fig. 3K), whereas ENO1 knockdown had the opposite effect (Fig. S3E). The degradation of RRM2 upon ENO1 knockdown was reversed by the proteasome inhibitor MG132 but not by CHL (Fig. S3F), indicating that ENO1 knockdown promotes proteasome-mediated degradation of RRM2. Further examination of RRM2 ubiquitination levels showed that overexpression of ENO1 reduced RRM2 ubiquitination while knockdown increased it (Fig. 3l). Collectively,ENO1 enhances RRM2 protein stability by decreasing its ubiquitination and proteasomal degradation.

### STUB1 acts as an E3 ubiquitin ligase for RRM2 to promote its degradation

**Fig. 4:**
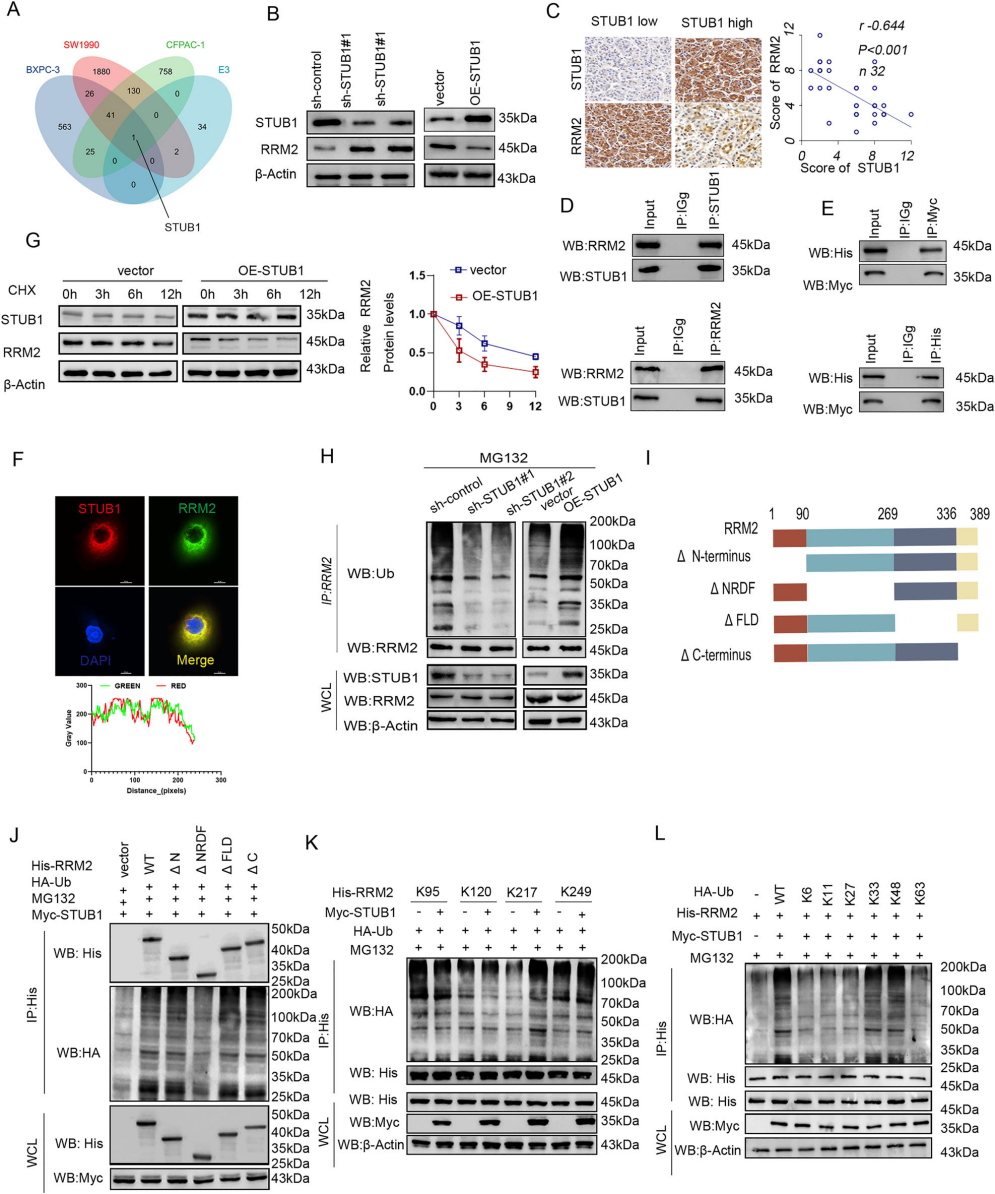
STUB1 acts as an E3 ubiquitin ligase for RRM2 to promote its degradation. **A** Intersection of significantly downregulated genes in gemcitabine-resistant pancreatic cancer cell lines from GEO datasets GSE140077 (Log2 FC > 1, *P* < 0.01) and GSE154909 (Log2 FC > 1, *P* < 0.01) with predicted E3 ligases of RRM2. **B** Knockdown of STUB1 in PANC-1 cells and overexpression of STUB1 in SW1990 cells, followed by WB detection of RRM2 protein levels. **C** IHC of STUB1 and RRM2 in pancreatic cancer tissues and their correlation. Statistical test: Spearman’s rank correlation. **D** Endogenous STUB1 or RRM2 precipitation in PANC-1 cell lysates using specific antibodies, followed by WB detection. **E** Co-IP assay in HEK293T cells transfected with Myc-STUB1 and His-RRM2 plasmids. His and Myc antibodies were used for protein precipitation and WB detection. **F** Confocal images showing co-localization of STUB1 (red) and RRM2 (green) in PANC-1 cells, with DAPI staining for nuclei (blue). **G** CHX chase analysis of RRM2 in SW1990 cells overexpressing STUB1. Each data point represents the average intensity of protein blots from three replicate experiments at each time point. **H** PANC-1 cells were transfected with the indicated sh-RNA (left panel), and SW1990 cells were transfected with the STUB1 plasmid (right panel). Cell lysates were subjected to IP with an RRM2 antibody, followed by WB detection. Cells were treated with 20 μM MG132 for 8 h before harvest. **I** Schematic diagram of full-length RRM2 and various truncation mutants. **J** Ubiquitination analysis in HEK293T cells co-transfected with HA-Ub, Myc-STUB1, and His-tagged RRM2 and its various deletion mutants, treated with 20 μM MG132 for 8 h. **K** Ubiquitination analysis in HEK293T cells co-transfected with HA-Ub, Myc-STUB1, and specified mutant His-RRM2, treated with 20 μM MG132 for 8 h. **L** Ubiquitination analysis in HEK293T cells co-transfected with Myc-STUB1, His-RRM2, and specified mutant HA-Ub, treated with 20 μM MG132 for 8 h. All in vitro experiments were carried out in three replicates.

Since ENO1 is not an E3 ubiquitin ligase and cannot directly mediate the ubiquitination and degradation of RRM2, we hypothesized that other E3 ligases are involved in the regulation of RRM2 stability mediated by ENO1. We utilized Ubibrowser (http://ubibrowser.bio-it.cn/ubibrowser/) to predict E3 ligases for RRM2 and combined this with transcriptional data from the Gene Expression Omnibus (GEO) database, which included gemcitabine-resistant and gemcitabine-sensitive cell types (GSE79953 and GSE140077). We discovered a significant decrease in STUB1 expression in resistant cell lines (Fig. 4A). First, we explored the impact of STUB1 on gemcitabine resistance in pancreatic cancer. STUB1 knockdown significantly decreased the sensitivity of pancreatic cancer cells to gemcitabine, while RRM2 knockdown restored the sensitivity. Conversely, Overexpression of STUB1 increases sensitivity, while RRM2 overexpression reverses it (Fig. S4A, B). Next, we investigated the impact of STUB1 on the expression of RRM2. Our findings indicated that STUB1 overexpression decreased the expression of RRM2 protein, while STUB1 knockdown increased it (Fig. 4B) without altering RRM2 mRNA levels (Fig. S4 C). Additionally, IHC revealed a moderate negative correlation between STUB1 and RRM2 expression in pancreatic cancer tissues (Fig. 4C). Furthermore, we confirmed the interaction between RRM2 and STUB1 through co-IP experiments (Fig. 4D, E), and immunofluorescence co-localization revealed that STUB1 and RRM2 are primarily distributed in the cytoplasm (Fig. 4F). Overexpression of STUB1 significantly reduced the protein stability of RRM2 (Fig. 4G), and markedly increased its ubiquitination level. Conversely, STUB1 knockdown decreased the ubiquitination level of RRM2 (Fig. 4H). Collectively, STUB1 indeed acts as an E3 ligase for RRM2.

To identify the specific sites of STUB1-mediated ubiquitination of RRM2, we constructed a series of truncated mutants based on the structural domains of RRM2 (Fig. 4I). Ubiquitination assays revealed that the absence of the NRDF domain abolished the effect of STUB1 on RRM2ubiquitination(Fig. 4J).UsingSUMOplo (http://www.abgent.com/sumoplot), we predicted potential ubiquitination sites within the NRDF domain and identified lysines 95, 120, 217, and 249 as candidates. Subsequent construction and analysis of the mutant proteins revealed that STUB1 predominantly targeted RRM2 for ubiquitination at lysine 217 (Fig. 4K). Mutation of this residue also significantly enhanced RRM2 protein stability (Fig. S4D).Given that distinct ubiquitin chain linkages can modulate protein properties such as stability, trafficking, and enzymatic function,we mutated lysine residues within the ubiquitin chain. Our findings indicate that STUB1 facilitates the attachment of K33 and K48 ubiquitin chains to RRM2 (Fig. 4L). Therefore, we conclude that STUB1 regulates K33- and K48-linked ubiquitination of RRM2 at lysine 217 (K217), thereby modulating RRM2 expression.

### ENO1 Interference with STUB1-RRM2 Interaction

**Fig. 5:**
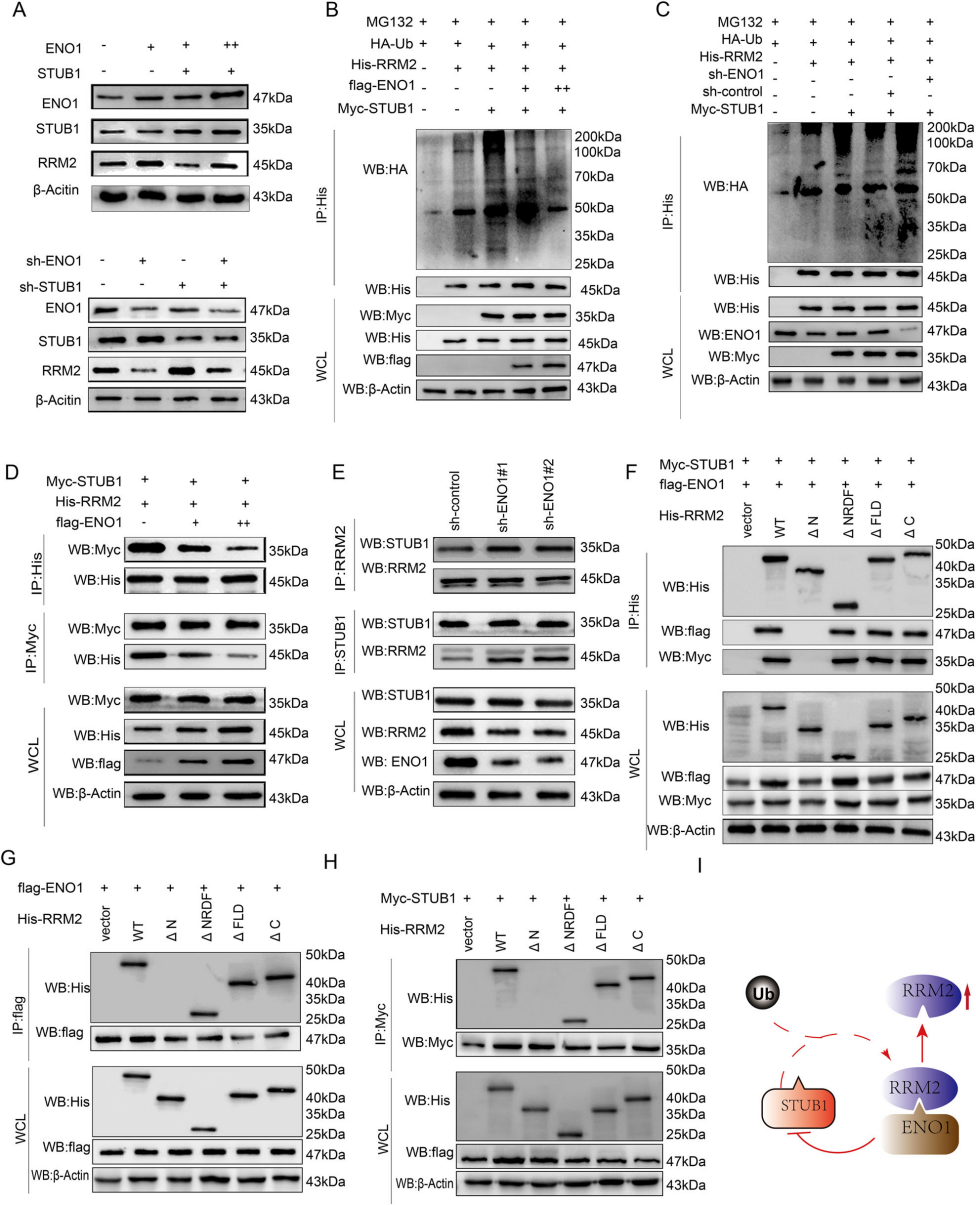
ENO1 Interference with STUB1-RRM2 Interaction. **A** In PANC-1 cells, different doses of ENO1 were co-transfected with a fixed amount of STUB1 (top), and in SW1990 cells, ENO1 and STUB1 were knocked down (bottom). The impact on RRM2 expression was detected using WB. **B** Co-transfection of Myc-STUB1, His-RRM2, HA-Ub, and different amounts of flag-ENO1 in HEK293T cells, treated with 20 μM MG132 for 8 h, followed by ubiquitination analysis of His-RRM2. **C** Co-transfection of Myc-STUB1, His-RRM2, HA-Ub, and sh-ENO1 in HEK293T cells, treated with 20 μM MG132 for 8 hours, followed by ubiquitination analysis of His-RRM2. **D** Co-transfection of Myc-STUB1, His-RRM2, and different amounts of flag-ENO1 in HEK293T cells, with His and Myc antibodies used for protein precipitation and WB detection. **E** Transfection of sh-ENO1 in PANC-1 cells, with RRM2 and STUB1 antibodies used for protein precipitation and WB detection. **F** Co-transfection of Myc-STUB1, flag-ENO1, and specified His-RRM2 truncation mutants in HEK293T cells, with Anti-His used for protein precipitation and WB detection. **G** Co-transfection of flag-ENO1 and specified His-RRM2 truncation mutants in HEK293T cells, with flag used for protein precipitation and WB detection. **H** Co-transfection of Myc-STUB1 and specified His-RRM2 truncation mutants in HEK293T cells, with flag used for protein precipitation and WB detection. **I** Schematic diagram of ENO1 and STUB1 competitively binding to RRM2 to inhibit RRM2 ubiquitination and degradation.All in vitro experiments were carried out in three replicates.

We explored the possibility that ENO1 and STUB1 have a joint regulatory effect on RRM2, given their contrasting impacts on its stability and ubiquitination. Our findings demonstrated that ENO1 overexpression can ameliorate the STUB1-induced reduction in RRM2 protein levels in a dose-dependent manner. Conversely, ENO1 knockdown reversed the STUB2-induced increase in RRM2 protein levels (Fig. 5A). Ubiquitination assays also revealed that ENO1 significantly reduces the ubiquitination levels of RRM2 mediated by STUB1. Increasing ENO1 expression further reduced RRM2 ubiquitination levels (Fig. 5B), whereas ENO1 knockdown reversed the decrease in RRM2 ubiquitination levels caused by STUB1 knockdown (Fig. 5C). Consequently, we hypothesized that ENO1 may competitively bind to RRM2, thereby diminishing the STUB1-RRM2 interaction and affecting RRM2 ubiquitination. To validate this hypothesis, we conducted co-IP assays to examine the effect of ENO1 on the binding of STUB1 to RRM2. ENO1 overexpression reduced the STUB1-RRM2 complex in a dose-dependent manner (Fig. 5D), whereas ENO1 knockdown strengthened this interaction (Fig. 5E). To investigate the competitive binding of ENO1 and STUB1 to RRM2, we performed co-IP analyses using RRM2 truncation mutants in the presence of STUB1 and ENO1, revealing that both proteins could concurrently bind to the N-terminus of RRM2 (Fig. 5F–H). Overall, ENO1 inhibits STUB1-mediated polyubiquitination and degradation of RRM2 by competing with STUB1 for binding to the N-terminus of RRM2.

### ENO1-RRM2 axis modulate deoxycytidine synthesis and promote gemcitabine resistance in pancreatic cancer

**Fig. 6:**
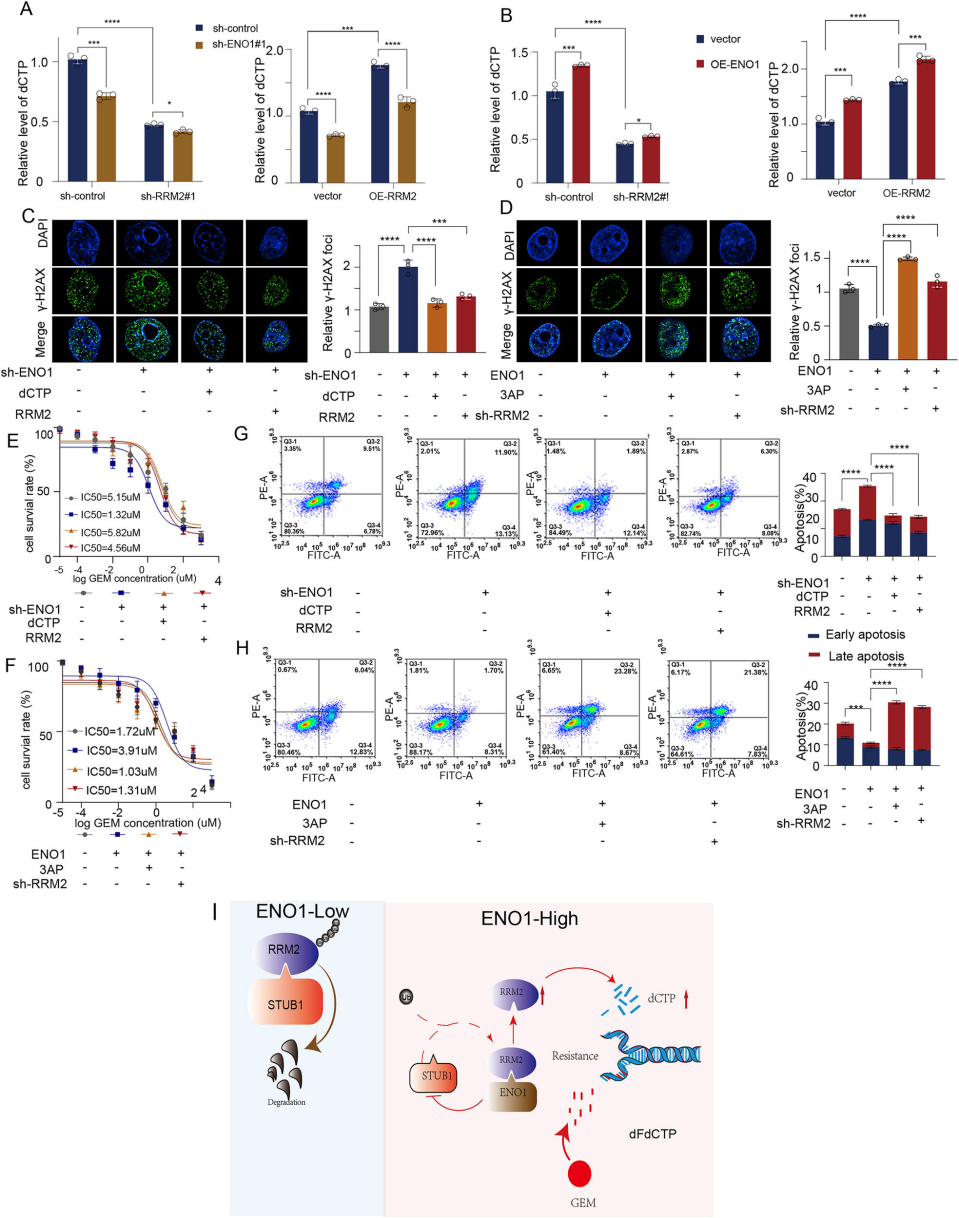
ENO1-RRM2 axis modulate deoxycytidine synthesis and promote gemcitabine resistance in pancreatic cancer. In PANC-1 cells, ENO1 was knocked down (**A**), and in SW1990 cells, ENO1 was overexpressed (**B**). Subsequently, RRM2 was either knocked down or overexpressed, and dCTP levels were measured. analyzed using a two-tailed Student’s t-test. **C**, **D**. Representative fluorescence images and quantitative statistical analysis of γ-H2AX foci in PANC-1 cells after knocking down ENO1 and adding dCTP or overexpressing RRM2, induced by gemcitabine (5 μM, 24 hours) (**C**). In SW1990 cells, after overexpressing ENO1 and adding RRM2 inhibitor (3AP) or knocking down RRM2, induced by gemcitabine (2 μM, 24 h) (**D**). Data are analyzed using a two-tailed Student’s t-test. **E**, **F**. CCK8 assay to detect gemcitabine sensitivity in PANC-1 cells after knocking down ENO1 and adding dCTP or overexpressing RRM2 (**E**), and in SW1990 cells after overexpressing ENO1 and adding RRM2 inhibitor (3AP) or knocking down RRM2 (**F**).analyzed using a two-tailed Student’s t-test. **G**, **H**. Apoptosis rate and corresponding statistical graph in PANC-1 cells after knocking down ENO1 and adding dCTP or overexpressing RRM2, induced by gemcitabine (5 μM, 24 h) (**G**). In SW1990 cells, after overexpressing ENO1 and adding RRM2 inhibitor (3AP) or knocking down RRM2, induced by gemcitabine (2 μM, 24 h) (**H**). Data are analyzed using a two-tailed Student’s t-test. **I**. Schematic diagram of the mechanism by which ENO1-mediated upregulation of RRM2 promotes gemcitabine resistance in pancreatic cancer.All in vitro experiments were carried out in three replicates.

Given that RRM2 is a key rate-limiting enzyme in the synthesis of deoxypyrimidines [22, 32], and considering that ENO1 can increase RRM2 protein levels, we further explored whether ENO1-mediated upregulation of RRM2 affects deoxypyrimidine synthesis in pancreatic cancer cells. We found that knockdown of ENO1 or RRM2 significantly reduced intracellular levels of dCDP and dCTP, while overexpression of ENO1 or RRM2 markedly increased these levels. ENO1 overexpression could reverse the decrease in dCDP and dCTP levels caused by RRM2 knockdown and vice versa. Additionally, ENO1 overexpression in RRM2-overexpressing cells further increased dCDP and dCTP levels, while ENO1 knockdown in RRM2-knockdown cells further decreased these levels (Figs. 6A, B and S5A, B). These findings indicate that ENO1 regulates deoxypyrimidine synthesis in pancreatic cancer cells via RRM2.

Deoxypyrimidines are known to compete with gemcitabine for DNA binding, reducing reactive oxygen species (ROS) production and replication stress [32]. Our findings revealed that overexpression of RRM2 or exogenous addition of dCTP in ENO1-knockdown cells significantly reduced ROS levels (Fig. S5C) and replication stress (Fig. 6C), while RRM2 knockdown or treatment with the RRM2 inhibitor 3AP reversed the reduction in ROS levels (Fig. S4D) and replication stress caused by ENO1 overexpression (Fig. 6D).

Finally, we examined whether ENO1 promotes gemcitabine resistance in pancreatic cancer cells by mediating deoxypyrimidine synthesis via RRM2. RRM2 overexpression or addition of dCTP rescued the change in IC50 caused by ENO1 knockdown(Fig. 6E). Similarly, RRM2 knockdown or addition of 3AP significantly reduced the IC50 of gemcitabine in ENO1-overexpressing cells(Fig. 6F). Additionally, EdU, CCK8, and flow cytometry assays showed that overexpression of RRM2 or addition of dCTP reversed the changes in growth rate and apoptosis of pancreatic cancer cells treated with gemcitabine due to ENO1 knockdown(Figs. 6G and S5E, G), whereas RRM2 knockdown or addition of 3AP rescued the changes caused by ENO1 overexpression(Figs. 6H and S5F, H). These findings demonstrate that ENO1 modulates the synthesis of deoxypyrimidines in pancreatic cancer cells through RRM2, thereby increasing their resistance to gemcitabine.

## Discussion

Gemcitabine is a first-line treatment for pancreatic cancer, but its clinical efficacy is limited owing to widespread drug resistance [3, 33]. However, there is currently a lack of clinical drugs to effectively overcome gemcitabine resistance [18]. Therefore, identifying new targets to improve gemcitabine resistance in pancreatic cancer is crucial. In this study, Our findings indicate that high expression levels of ENO1 are closely associated with gemcitabine resistance in pancreatic cancer, and we have elucidated the underlying molecular mechanisms. These insights offer new therapeutic strategies to improve gemcitabine resistance in pancreatic cancer.

Numerous studies have identified several mechanisms of gemcitabine resistance in pancreatic cancer, such as deficiencies in deoxycytidine kinase (DCK) activity limiting gemcitabine activation [34], increased deoxycytidine deaminase (CDA) activity leading to rapid metabolism of gemcitabine, and reduced nucleoside transporter activity restricting gemcitabine entry into cells [8]. However, these mechanisms do not fully account for gemcitabine resistance in pancreatic cancer cells. Our study revealed that ENO1 significantly enhances gemcitabine resistance in pancreatic cancer. Through strict inclusion and exclusion criteria, we collected clinical data from patients with pancreatic cancer, consistent with previous studies confirming that ENO1 expression is closely related to patient prognosis [35]. We also found that high ENO1 expression correlates with higher tumor stages, lymph node metastasis, poorer differentiation, and increased portal vein invasion rates. These findings suggest that ENO1 expression is closely related to the malignancy and progression of pancreatic cancer, indicating its potential as a marker for pancreatic cancer [36, 37]. Additionally, we found that high ENO1 expression is an independent risk factor for recurrence within 1 year in patients with pancreatic cancer who underwent radical surgery and postoperative gemcitabine-based adjuvant chemotherapy. This finding highlights the strong correlation between ENO1 expression and gemcitabine resistance in pancreatic cancer, suggesting that high ENO1 expression may identify patients less likely to benefit from gemcitabine monotherapy, enabling more personalized treatment strategies.

Our in vivo and in vitro experiments showed that ENO1 knockdown sensitized pancreatic cancer cells to gemcitabine, while overexpression of ENO1 conferred resistance. Previous studies by Ma et al. indicated that ENO1 increases gemcitabine resistance in pancreatic cancer [38]. Our experimental and clinical data provide substantial evidence that ENO1 modulates gemcitabine resistance in pancreatic cancer. ENO1, as a glycolytic enzyme, has been studied for its high expression, which affects cell glycolytic metabolism, as well as cell proliferation, migration, and differentiation [39, 40]. Notably, our study found that ENO1’s role in increasing gemcitabine resistance in pancreatic cancer is independent of its glycolytic enzymatic activity. As a multifunctional enzyme, an increasing number of studies have shown that ENO1’s role in tumor progression may not depend on its enzymatic activity [41], Sun et al. reported that ENO1, as an RNA-binding protein, regulates lipid synthesis and promotes liver cancer progression [29]. These findings and those from our study emphasize the need to explore the non-enzymatic functions of ENO1 as a potential therapeutic target.

ENO1 is highly expressed in various malignancies. However, the mechanism through which it promotes gemcitabine resistance in pancreatic cancer is not well understood. Ma et al. found that ENO1 interacts with CHKα to regulate phosphatidylcholine synthesis [42]. IP-MS and co-IP experiments in this study identified RRM2 as an ENO1-interacting protein. RRM2 is the primary regulatory component of ribonucleotide reductase (RR) activity and is involved in nucleotide metabolism [43]. In drug-resistant tumor cells, the RRM2 gene and its promoter region show significant amplification, leading to high transcription levels [26]. However, the regulatory mechanisms of RRM2 are quite complex, with previous studies mainly focusing on its transcriptional and translational regulation. Jiang et al. found that the UBE2T/RING1/p53 axis regulates RRM2 transcription and promotes gemcitabine resistance in pancreatic cancer [44]. While there have been few studies on the regulatory mechanisms controlling RRM2 stability, which are key mechanisms in many pathological processes. We identified a novel mechanism for regulating RRM2, revealing that ENO1 controls the degradation of RRM2 in a manner independent of its enzymatic activity. In the RRM2 protein complex, STUB1, an E3 ligase, is an RRM2-associated protein that increases ubiquitin chains on the K217 residue of RRM2, promoting its degradation. Zeng et al. reported that STUB1 increases the sensitivity of glioma cells to cisplatin by mediating the degradation of SMYD2 [45]. Furthermore, we found that ENO1 competes with STUB1 for binding to RRM2. As ENO1 expression increases, the binding of STUB1 to RRM2 decreases in a dose-dependent manner. Further studies revealed that ENO1 interacts with the N-terminus of RRM2 in the same binding region as STUB1, thereby inhibiting the interaction between RRM2 and STUB1 and eliminating STUB1-dependent polyubiquitination and degradation of RRM2. These findings emphasize the importance of ENO1 in regulating RRM2 protein levels.

Metabolic reprogramming is a hallmark of tumors, and pyrimidine metabolism is crucial for gemcitabine resistance in pancreatic cancer [46]. Targeting deoxypyrimidine synthesis has shown promising results for improving chemotherapy resistance in preclinical studies [47]. However, the regulation of deoxypyrimidine synthesis in cancer remains largely unexplored.We found that ENO1 significantly increases deoxypyrimidine synthesis in pancreatic cancer cells and that ENO1 and RRM2 have a synergistic effect on deoxypyrimidine synthesis. Similarly, further experiments demonstrated that the effect of ENO1 on gemcitabine resistance in pancreatic cancer cells depends on RRM2-mediated production of deoxycytidine. These findings indicate that ENO1 reprograms pyrimidine metabolism in pancreatic cancer cells, thereby promoting gemcitabine resistance.

Our current research indicates that ENO1 may serve as a critical target for overcoming gemcitabine resistance in pancreatic cancer. Studies have shown that monoclonal antibodies (mAbs) targeting ENO1 can inhibit the invasion and metastasis of pancreatic cancer cells. Additionally, anti-ENO1 mAbs can mediate complement-dependent cytotoxicity, thereby killing pancreatic cancer cells [48]. Combining the ENO1 vaccine with chemotherapeutic agents such as gemcitabine can further enhance the anti-tumor efficacy [49]. Similarly, the combination of the ENO1 vaccine with a PI3Kγ inhibitor can strengthen the immune response and reduce angiogenesis [50]. These findings collectively suggest that ENO1, as an important therapeutic target in pancreatic cancer, holds significant clinical potential. Future research should further explore the impact of ENO1 on the tumor microenvironment and advance its clinical applications.

This study high ENO1 expression increases deoxypyrimidine synthesis, thereby promoting gemcitabine resistance in PDAC. Targeting the ENO1-STUB1-RRM2 axis holds significant clinical implications for improving the sensitivity of pancreatic cancer cells to gemcitabine.

## Methods and materials

### Antibodies and reagents used in the experiment

The antibodies used in this experiment include: ENO1 (ab227978), RRM2 (ab172476), STUB1 (ab134064), and flag (ab205606), all procured from abcam. Additionally, HA (3724), Myc (2276), His (12698), ubiquitin (3936), HRP-conjugated anti-mouse IgG (7076), and HRP-conjugated anti-rabbit IgG (7074) were obtained from Cell Signaling Technology. Ki-67 (27309-1-AP), Caspase 3 (82707-13-RR), and β-ACTIN (20536-1-AP) were purchased from proteintech.

The pharmaceuticals tested in this study include gemcitabine (LY 188011), Z-Leu-Leu-Leu-al (MG132; HY-13259), cycloheximide (CHX; HY-12320), Chloroquine (CHL; HY-17589A), and Triapine (3-APPAN-811), all sourced from MedChemExpress (MCE). The drugs were dissolved in dimethyl sulfoxide (DMSO). Anti-HA magnetic beads (HY-K0201A), Anti-Flag Magnetic Beads (HY-K0207), Anti-His magnetic beads (HY-K0209), Anti-c-Myc magnetic beads (HY-K0206), and protein A/G magnetic beads (HY-K0202) were also acquired from MCE.

### Bioinformatics analysis

GEPIA2 (http://gepia2.cancer-pku.cn/#general) is a tool that integrates The Cancer Genome Atlas (TCGA) and Genotype-Tissue Expression (GTEx) to analyze gene expression in cancerous and non-cancerous specimens. We assessed the levels of ENO1 in pancreatic cancer and non-cancerous tissues, and based on the expression levels of ENO1, we examined the overall survival and disease-free survival rates in pancreatic cancer (PC). Microarray expression data from the GEO database (https://www.ncbi.nlm.nih.gov/geo/), including the GSE79953 and GSE140077 datasets, were analyzed to identify downregulated genes in drug-resistant pancreatic cancer cell lines. Statistical analysis was performed using the “limma,” “edgeR,” and “stats” packages, and data visualization was conducted with the “ggplot2” sofHuman Pancreatic Cancer Specimens Collected tware package.

### Human pancreatic cancer specimens collected

Between 2018 and 2023, a retrospective collection of paraffin-embedded pancreatic cancer specimens from patients who underwent radical surgery was conducted at the Affiliated Hospital of Guizhou Medical University. Corresponding clinical data and follow-up information were also gathered. In addition to these, we collected 90 cases of pancreatic cancer and adjacent tissue specimens stored in RNALater preservation solution, as well as 12 pairs of fresh pancreatic cancer and adjacent tissues. These were used to detect the mRNA and protein expression levels of ENO1. All tissue specimens were stained with hematoxylin and eosin (H&E) and were re-evaluated histologically by two independent pathologists.

### Immunohistochemistry(IHC)

Tissue sections were deparaffinized with xylene and rehydrated using a graded ethanol series. Samples were incubated with citrate buffer for antigen retrieval and then blocked with goat serum (Beyotime, C0265) to prevent non-specific binding. Subsequently, the corresponding primary antibodies were incubated overnight at 4°C, followed by incubation with the corresponding secondary antibodies for 2 hours at room temperature. Three randomly selected areas were evaluated for positively stained cells and signal intensity by two independent observers in a blinded manner. The scoring criteria were as follows: cell staining intensity was scored on a scale of 3 (negative=0, weakly positive=1, positive=2, strongly positive=3), and the proportion of positive cells was scored on a scale of 4 (<1% = 0, 1–25% = 1, 26–50% = 2, 51–75% = 3, >75% = 4). The immunohistochemistry score was obtained by multiplying these two scores.

### Cell culture

Human pancreatic cancer cell lines (MiaPaCa-2, Capan-1, PANC-1, BxPC-3, SW1990, AsPC-1, and HPDE cells) and HEK239T cells were sourced from the American Type Culture Collection (ATCC) and maintained at 37 °C in a 5% CO_2_ incubator. To ensure authenticity, all cell lines undergo short tandem repeat (STR) analysis for routine identification once a year. Additionally, mycoplasma contamination is detected using PCR every two months. All cell lines are used between passages 5 and 10. HPDE, PANC-1, and MiaPaCa-2 cells are cultured in DMEM (Gibco, 11320033) supplemented with 10% Fetal Bovine Serum (FBS) (Gibco, A5256701). Capan-1 cells are cultured in IMDM (Gibco, 12440061) containing 10% FBS. AsPC-1 and BxPC-3 cells are cultured in RPMI-1640 (Gibco, 11875093) medium with 10% FBS.

### RNA extraction and real-time quantitative polymerase chain reaction (qRT-PCR)

Total RNA was extracted from cells and tissues using TRIzol reagent (Invitrogen, 15596018CN) according to the manufacturer’s protocol. Complementary DNA (cDNA) was synthesized using the PrimeScript RT reagent kit (TaKaRa, RR047A) following the user guide. Gene expression levels were assessed using real-time fluorescent quantitative polymerase chain reaction (qRT-PCR). The mRNA levels of genes were calculated using the 2 − ΔΔCt method and normalized to the expression levels of GAPDH.Te Forward primer for ENO1(5′-GCCATTGATCGCGAGCACTC-3′), Reverse primer (5′-GAGACAGCCTGTAGTGTTGT-3′), Forward primer forGAPDH (5′-CCACAGTCCATGCCATCACTG-3’), Reverse primer (5′-GTCAGGTCCACCACTGACACG-3′). Te Forward primer for RRM2 (5′-TACCCAAACTGTAGTGATTC-3′)Reverse primer (5′-TCCCAGGACTTCCACTGATTCT-3′). Te Forward primer for STUB1(5′-ATTCCCAAGTGTCGTCATGG-3′)Reverse primer(5′-TTAACACCGATTTTAGGTGACG-3′)

### Western blot analysis

Proteins were extracted from cells and tissues using RIPA buffer (MCE, HY-K1001) and a protease inhibitor cocktail (MCE, HY-K0010) according to the manufacturer’s instructions. Protein concentrations were determined using a bicinchoninic acid (BCA) assay kit (Beyotime, P0010). Proteins were separated by sodium dodecyl sulfate polyacrylamide gel electrophoresis (SDS-PAGE) and transferred onto PVDF membranes. After blocking with 5% skim milk for 2 h, the membranes were incubated with the corresponding primary antibodies overnight at 4 °C, followed by incubation with secondary antibodies at room temperature for 2 h. Protein expression levels were detected using an ECL chemiluminescent solution (Merck, WBULS0500) and a ChemiDoc™ imaging system (Bio-Rad, USA).

### Immunoprecipitation, silver staining, and mass spectrometry

NP40 lysis buffer (Beyotime,p0013F)with protease inhibitors was added to cells. Cells were lysed on ice for 30 min and then centrifuged at 12,000 rpm for 10 min to collect the clear protein supernatant. The supernatant was incubated with the corresponding primary antibody overnight at 4 °C. Subsequently, Protein A + G magnetic beads (Beyotime, P2108) were added to the mixture according to the manufacturer’s instructions and incubated at 4 °C for an additional 4 hours with rotation. After complete centrifugation and washing, the beads were collected and boiled to elute the proteins. Finally, the resulting samples were subjected to western blotting, silver staining, or mass spectrometry (MS) analysis. Silver staining was performed using a Silver Staining Detection Kit (Thermo Fisher, 24600), following the manufacturer’s protocol. MS analysis was conducted at Gene Apsaras Company (Wuhan, Hubei, China).

### CCK-8 assay

Cells (3,000 per well) were seeded into 96-well plates and allowed to adhere completely. Following this, DMSO or a predetermined concentration of gemcitabine was added according to the experimental schedule, and the cells were cultured for 0, 24, 48, and 72 hours. Subsequently, a mixed solution (110 μl, consisting of 10 μl CCK-8 reagent + 100 μl complete culture medium) was added according to the manufacturer’s instructions (MCE, HY-K0301). After a 2-hour incubation, the absorbance at 450 nm was measured using a plate reader (Tecan, Austria). Each group had five replicate wells, and three independent replicate experiments were conducted. The concentration of GEM that reduced cell viability by 50% was determined by fitting a nonlinear least-squares curve to the dose-response curve. This value was then used to obtain the half-maximal inhibitory concentration (IC50).

### Colony formation assay

Pancreatic cancer (PC) cells were plated in 6-well plates at a density of 1000 cells per well and incubated with DMSO or a specific concentration of gemcitabine for 2 weeks according to the experimental protocol. Cells were fixed with 4% paraformaldehyde for 20 min. After washing with phosphate-buffered saline (PBS), cells were stained with a 0.25% crystal violet solution for 20 min. Finally, the culture plates were photographed.

### EdU assay

3 × 10^4^ cells were seeded into 24-well plates with coverslips and incubated with DMSO or a specific concentration of gemcitabine for 24 h according to the experimental protocol. EdU solution (Servicebio, G4102-100T) was added and incubated for 2 h following the manufacturer’s instructions. Cells were fixed with 4% paraformaldehyde for 20 minutes and washed with PBS. After permeabilization with 0.3% Triton X-100 for 10 min, EdU was detected with an IF 555 staining kit, and cell nuclei were stained with DAPI. An Olympus FSX100 microscope was used to capture images.

### Cell flow cytometry analysis

Cells were plated in 6-well plates and incubated with DMSO or a specific concentration of gemcitabine for 48 hours. After trypsinization and centrifugation, cells were fixed with prechilled 70% ethanol at −20 °C for 2 h. RNase A and PI were added according to the Cell Cycle and Apoptosis Detection Kit (Beyotime, C1052) instructions and incubated for 30 minutes. For apoptosis detection, the Annexin V/PE Apoptosis Detection Kit (Beyotime, C1065) was used to measure the apoptosis rate, with 195 μl Annexin V-PE binding solution incubated according to the instructions. Then, 5 μl Annexin V-PE was added and incubated for 20 min in the dark. Analysis was performed using a Summit5.2 (Beckman Coulter, USA).

### Immunofluorescence assay

PC cells were seeded onto 24-well plates with coverslips and cultured for 24 hours. Cells were fixed with 4% paraformaldehyde and permeabilized with 0.5% Triton X-100 solution. Nonspecific binding sites were blocked with 5% bovine serum albumin. Primary antibodies were incubated at 4 °C for 12 h. After washing with PBS, cells were incubated with corresponding fluorescent secondary antibodies in the dark at room temperature for 2 h. After PBS washing, cell nuclei were stained with DAPI. A fluorescence microscope was used to detect the expression of target proteins.

### Detection of Dctp dCDP

Treated pancreatic cancer cells were cultured in T75 flasks until they reached approximately 80% confluence. Cells were harvested with 4 ml of 80% methanol on dry ice and sonicated for 5 min. After centrifugation at 15,000 × *g* for 20 min, the supernatant was collected and mixed with an equal volume of 2:1 H2O/CHCl3 solution and vortexed for 30 s. Metabolite extracts were dried under nitrogen using an N-EVAP. Then, 50% acetonitrile (ACN) was added to the samples and vortexed for 30 s. Analysis was performed using liquid chromatography-tandem mass spectrometry (LC-MS/MS) to determine the relative abundance of intracellular Dctp dCDP.

### ROS detection

Cells were washed twice with cold PBS and then stained with the fluorescent dye 2’,7’-dichlorodihydrofluorescein diacetate (H2DCF-DA; Sigma-Aldrich, 35845) at 37 °C for 30 min. Stained cells were washed, trypsinized, and resuspended in PBS. Fluorescence intensity was measured using a Varioskan LUX multimode plate reader (Thermo Fisher Scientific).

### Animal experiment

A total of 35 female BALB/c nude mice, 6 weeks old, were randomly divided into 7 groups. The corresponding stably transfected cells (2 × 10^6^ cells/mouse) were injected subcutaneously into the right axillary region of the mice. When the tumor volume reached approximately 100 mm^3^, gemcitabine or saline (intraperitoneal injection at 50 mg/kg/week) was administered according to the experimental plan. Tumor size was monitored every 4 days using calipers until euthanasia. The tumors were then excised, weighed, fixed in 4% paraformaldehyde, and embedded in paraffin for immunohistochemistry (IHC) analysis.

### Statistical analysis

Statistical analyses were performed using SPSS (version 26.0, IBM, USA) and GraphPad Prism (version 9.03, GraphPad, USA). The chi-square test was used to assess the correlation between ENO1 expression and clinical pathological characteristics. For skewed distribution data, the median (interquartile range) was used. Comparisons between two groups were made using the Student’s t-test (two-tailed), and comparisons among multiple groups were made using analysis of variance (ANOVA). Survival analysis was conducted using the Kaplan–Meier method, and differences between groups were analyzed using the Log-rank test. The Cox proportional hazards model was used for multivariable analysis to explore the relationship between ENO1 expression and survival in pancreatic cancer patients. Multivariable logistic regression analysis was used to investigate the relationship between ENO1 and gemcitabine resistance in pancreatic cancer. A significance level of *P* < 0.05 was considered to indicate a statistically significant difference.

#### Ethics approval and consent to participate

This study was approved and consented by the Human Ethics Committee of the Affiliated Hospital of Guizhou Medical University and the Animal Ethics Committee of Guizhou Medical University. All animal care procedures and experiments were performed according to the guidelines of the Institutional Animal Care and Use Committee (IACUC) guidelines. Since the clinical samples involved in this study were retrospectively collected, there is no need to sign an informed consent form.

## Supplementary information

Supplemental material

Original Data

## Data Availability

The original dataset for this study can be obtained by contacting the corresponding author, and all analyzed data are included in the article and additional files.
