# Supplementary material for: ENO1-mediated deoxycytidine synthesis and gemcitabine resistance by stabilizing RRM2 in pancreatic cancer
Source: Cell Death Dis. 2025 Dec 27;17(1):139. doi: 10.1038/s41419-025-08061-6 (PMC12847938; doi:10.1038/s41419-025-08061-6)
Supplement: Supplementary file 1 — Supplemental material [file 41419_2025_8061_MOESM1_ESM.docx]

Table S1 Relationship between ENO1 and clinical baseline characteristics of PC patients(chi-square test))

| Variable | ENO1 low | ENO1 high | P value |
| --- | --- | --- | --- |
| Age |  |  | 0.077 |
| ≤60y | 27 | 33 |  |
| >60y | 37 | 27 |  |
| Gender  （M/F） | 31/33 | 28/32 | 0.50 |
| CA199(IU) |  |  | 0.016 |
| ≤100 | 48 | 33 |  |
| ＞100 | 16 | 27 |  |
| Tumor location |  |  | 0.575 |
| Head | 47 | 46 |  |
| Body and tail | 17 | 14 |  |
| Tumor size |  |  | <0.01 |
| ≤2cm | 55 | 35 |  |
| ＞2cm | 9 | 25 |  |
| N stage |  |  | <0.01 |
| N0 | 51 | 25 |  |
| N1 | 10 | 25 |  |
| N2 | 3 | 10 |  |
| neural invasion |  |  | 0.072 |
| yes | 46 | 50 |  |
| NO | 18 | 10 |  |
| Pv invasion |  |  | 0.062 |
| YES | 20 | 29 |  |
| NO | 44 | 31 |  |
| tumor differentiation |  |  | 0.050 |
| high | 15 | 7 |  |
| middle | 21 | 18 |  |
| low | 28 | 35 |  |
| AJCC stage |  |  | <0.001 |
| I | 51 | 23 |  |
| II | 10 | 26 |  |
| III | 3 | 11 |  |
| GEM treatment |  |  | 0.823 |
| YES | 52 | 48 |  |
| NO | 12 | 12 |  |

Table S2 Univariate and multivariate COX risk regression model analysis for OS and RFS

| Variable | OS | | | |  |
| --- | --- | --- | --- | --- | --- |
|  | univariate analysis | | multivariate analysis | |  |
|  | HR(95%CI) | Pvalue | HR(95%CI) | P value |  |
| Age（y）  ＜60 VS ≥60 | 0.99（0.63-1.58） | 0.99 |  |  |  |
| Gender  Male vs female | 1.31（0.82-2.87） | 0.25 |  |  |  |
| CA199(IU)  100 vs ≥100 | 1.86（1.15-2.99） | 0.01 | 1.09(0.64-1.85) | 0.75 |  |
| Tumor location  Head VS Body and tail | 1.58（0.91-2.76） | 0.11 |  |  |  |
| Tumor size(cm)  ＜2 VS ≥2 | 1.65（1.28-2.13） | 0.01 | 1.65(0.79-1.62) | 0.49 |  |
| lymph node metastasis  NO VS YES | 2.63（1.65-4.18） | <0.01 | 2.12(1.13-3.98) | 0.02 |  |
| neural invasion  NO VS YES | 1.76（0.92-3.36） | 0.09 |  |  |  |
| Pv invasion  NO VS YES | 2.62(1.65-4.17) | <0.01 | 2.11(1.20-3.72) | 0.01 |  |
| tumor differentiation  High VS middle&low | 2.67（1.67-4.37） | <0.01 | 2.28(1.36-3.84) | <0.01 |  |
| ENO1 expresion  Low VS High | 2.05(1.29-3.27) | <0.01 | 1.45(0.89-2.38) | 0.14 |  |

| Variable |  | DFS | | | |
| --- | --- | --- | --- | --- | --- |
|  |  | univariate analysis | | multivariate analysis | |
|  |  | HR(95%CI) | P value | HR(95%CI) | P value |
| Age（y）  ＜60 VS ≥60 |  | 1.38(0.91-2.07) | 0.12 |  |  |
| Gender  Male vs female |  | 0.99(0.66-1.49) | 0.95 |  |  |
| CA199(IU)  100 vs ≥100 |  | 1.83(1.19-2.81) | <0.01 | 1.06(0.66-1.69) | 0.82 |
| Tumor location  Head VS Body and tail |  | 1.45(0.90-2.34) | 0.13 |  |  |
| Tumor size(cm)  ＜2 VS ≥2 |  | 2.25(1.44-3.50） | <0.01 | 0.69(0.36-1.31) | 0.25 |
| lymph node metastasis  NO VS YES |  | 2.80(1.84-4.23) | <0.01 | 1.79(0.99-3.25) | 0.06 |
| neural invasion  NO VS YES |  | 1.71(1.01-2.91） | 0.047 | 1.03(0.56-1.89) | 0.93 |
| Pv invasion  NO VS YES |  | 2.7（1.76-4.13） | <0.01 | 1.87(1.07-3.29) | 0.03 |
| tumor differentiation  High VS middle&low |  | 2.4（1.73-3.31） | <0.01 | 2.04(1.31-3.18) | <0.01 |
| ENO1 expresion  Low VS High |  | 2.26(1.50-3.41) | <0.01 | 1.91(1.2-3.06) | <0.01 |

Table S3 Multivariate logistic regression model analysis for gemcitabine resistance in pancreatic cancer patients.

| Variable | gemcitabine resistance | | | |  |
| --- | --- | --- | --- | --- | --- |
|  | univariate analysis | | multivariate analysis | |  |
|  | OR(95%CI) | Pvalue | OR(95%CI) | Pvalue |  |
| Age（y）  ＜60 VS ≥60 | 1.04（0.5-2.18） | 0.91 |  |  |  |
| Gender  Male vs female | 1.53 (0.73-3.21） | 0.25 |  |  |  |
| CA199(IU)  100 vs ≥100 | 2.81（1.3-6.09） | 0.01 | 1.69(0.66-4.28) | 0.27 |  |
| Tumor location  Head VS Body and tail | 2.05（0.83-5.03） | 0.12 |  |  |  |
| Tumor size(cm)  ＜2 VS ≥2 | 2.33（1.04-5.24） | 0.04 | 0.46(0.13-1.68) | 0.24 |  |
| lymph node metastasis  NO VS YES | 3.81(1.75-8.27) | <0.01 | 2.22(0.71-6.92) | 0.17 |  |
| neural invasion  NO VS YES | 2.25(0.83-6.10) | 0.09 |  |  |  |
| Pv invasion  NO VS YES | 2.64(1.24-5.64) | 0.01 | 1.93(0.69-5.42) | 0.21 |  |
| tumor differentiation  High VS middle&low | 2.25（1.06-4.80） | 0.03 | 1.60(0.67-3.81) | 0.29 |  |
| ENO1 expresion  Low VS High | 6.6(2.88-15.14) | <0.01 | 6.6(2.88-15.14) | <0.01 |  |


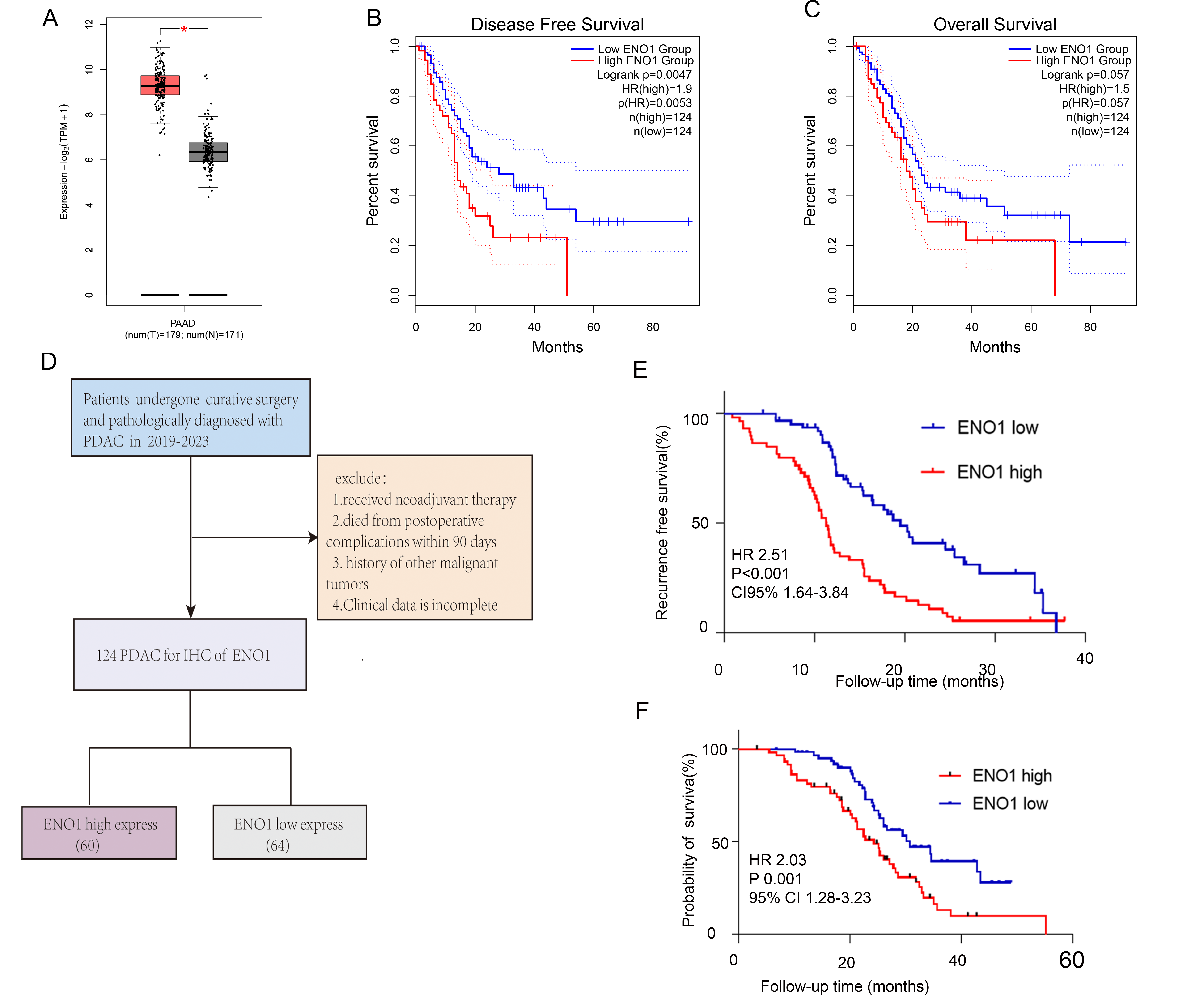
Fig. S1

ENO1 is highly expressed in pancreatic cancer and is associated with prognosis. **A.** Relative expression levels of ENO1 in pancreatic cancer and adjacent tissues based on the GEPIA2 database. **B** and **C**. Kaplan-Meier curves of disease-free survival and overall survival in pancreatic cancer patients based on ENO1 expression from the TCGA database. **D.** Flowchart for the retrospective inclusion of clinical pancreatic cancer patient data.  **E** and **F.** Kaplan-Meier curves of disease-free survival and overall survival in pancreatic cancer patients based on ENO1 expression from our hospital.


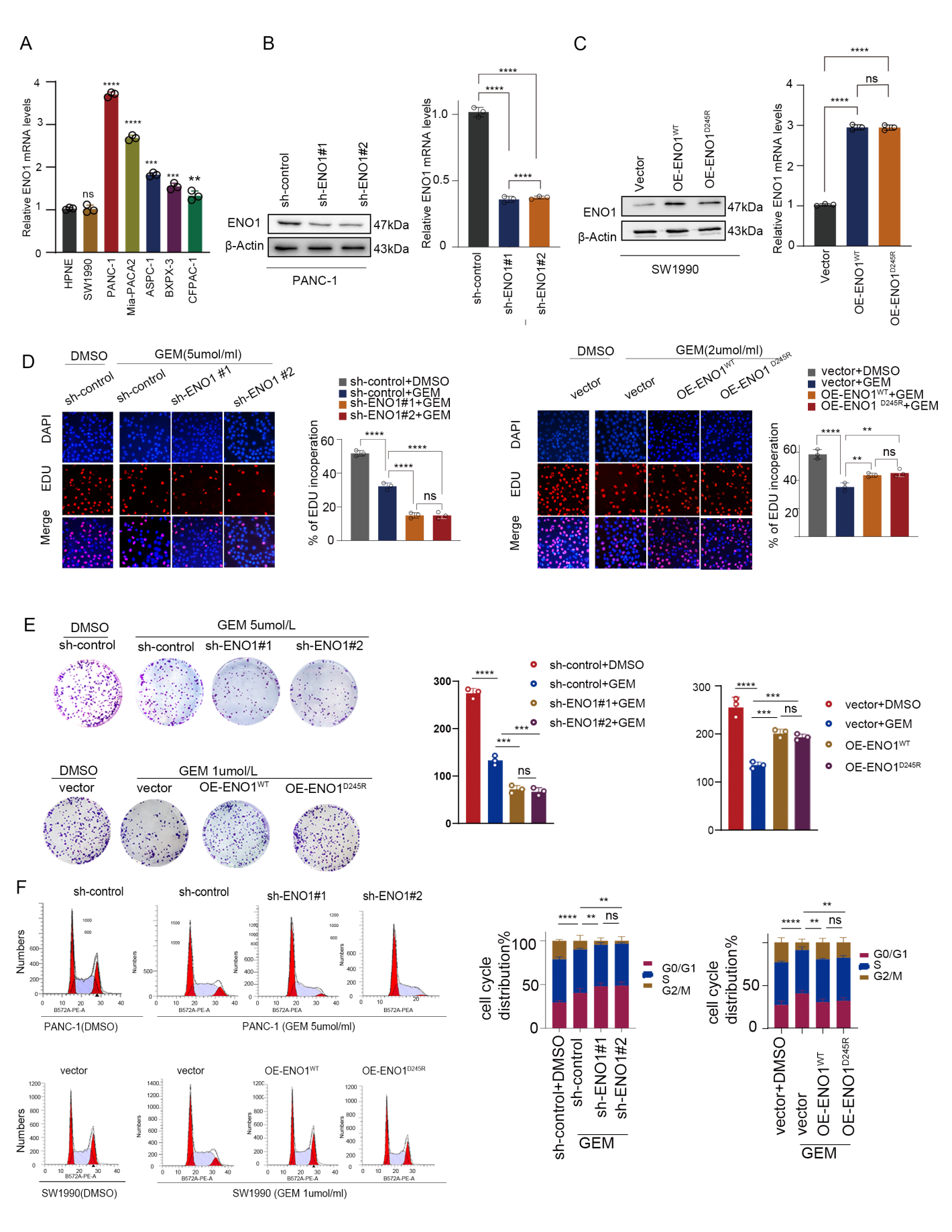


Fig. S2

ENO1 Mediates Gemcitabine Resistance in Pancreatic Cancer Independently of Enzymatic Activity **A.** qPCR was used to detect the expression levels of ENO1 mRNA in pancreatic epithelial and cancer cells. Statistical analysis ：ANOVA.  **B** and **C**. ENO1 was knocked down in PANC-1 cells and overexpressed as ENO1WT or ENO1D245 in SW1990 cells. The efficiency of knockdown and overexpression was detected using WB and qPCR. Statistical analysis : two-tailed Student's t-test. **D.** EDU assay was used to measure the proliferation efficiency of PANC-1 cells with ENO1 knockdown and SW1990 cells with ENO1^WT^ or ENO1^D245^R overexpression after treatment with DMSO or GEM (5 μmol/L GEM for PANC-1 cells, 2 μmol/L for SW1990). Statistical analysis : ANOVA. **E.** The colony formation ability of PANC-1 cells with ENO1 knockdown and SW1990 cells with ENO1WT or ENO1^D245R^ overexpression was assessed after treatment with DMSO or GEM (5 μmol/L GEM for PANC-1 cells, 2 μmol/L for SW1990). Statistical analysis : ANOVA. **F.** Cell cycle distribution of PANC-1 cells with ENO1 knockdown and SW1990 cells with ENO1WT or ENO1^D245R^ overexpression was assessed after treatment with DMSO or GEM (5 μmol/L GEM for PANC-1 cells, 2 μmol/L for SW1990). Statistical analysis : ANOVA. All in vitro experiments were carried out in three replicates


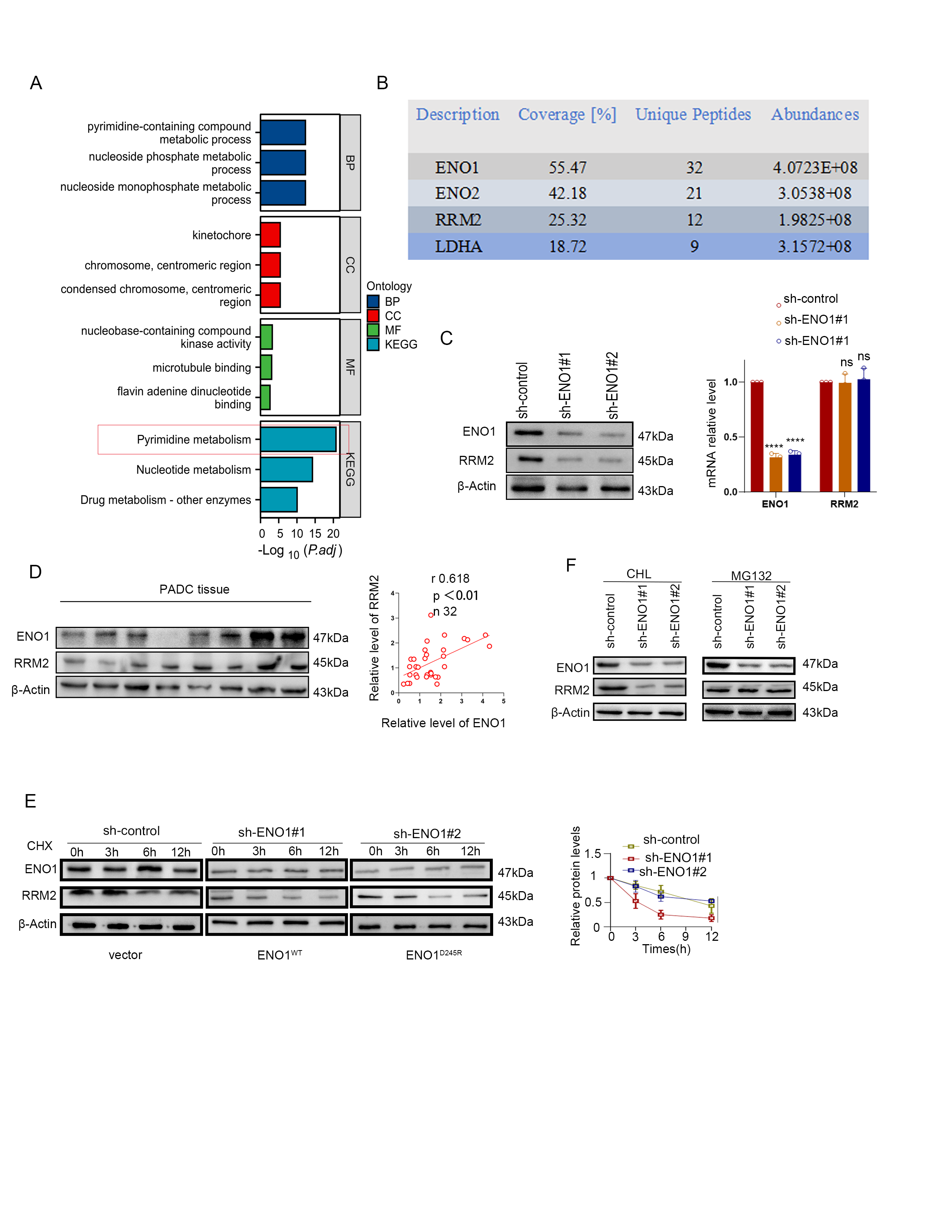


Fig. S3

ENO1 Binds to RRM2 to Enhance the Stability of the RRM2 Protein

**A.** BP, CC, MF, and KEGG pathway enrichment for proteins interacting with ENO1 as detected by IP-MS. **B.** Mass spectrometry detection of some proteins interacting with ENO1. **C.** After knocking down ENO1 in PANC-1 cells, changes in RRM2 protein and mRNA were detected using WB and qPCR.Statistical analysis : two-tailed Student's t-test. **D.** WB was used to quantify and correlate ENO1 and RRM2 protein levels in pancreatic cancer tissue samples. Each point represents the intensity of the protein blot, and statistical analysis was performed using Pearson's correlation. **E**. CHX chase analysis of RRM2 in PANC-1 cells with ENO1 knockdown. Cells expressing an empty vector served as a negative control. Each data point represents the average intensity of protein blots from three replicate experiments at each time point. **F.** After knocking down ENO1 in PANC-1 cells, proteasome inhibitor MG132 (20 μM, 8 h) or autophagy inhibitor chloroquine (CHL, 10 μM, 8 h) was added, and RRM2 was detected using WB.All in vitro experiments were carried out in three replicates


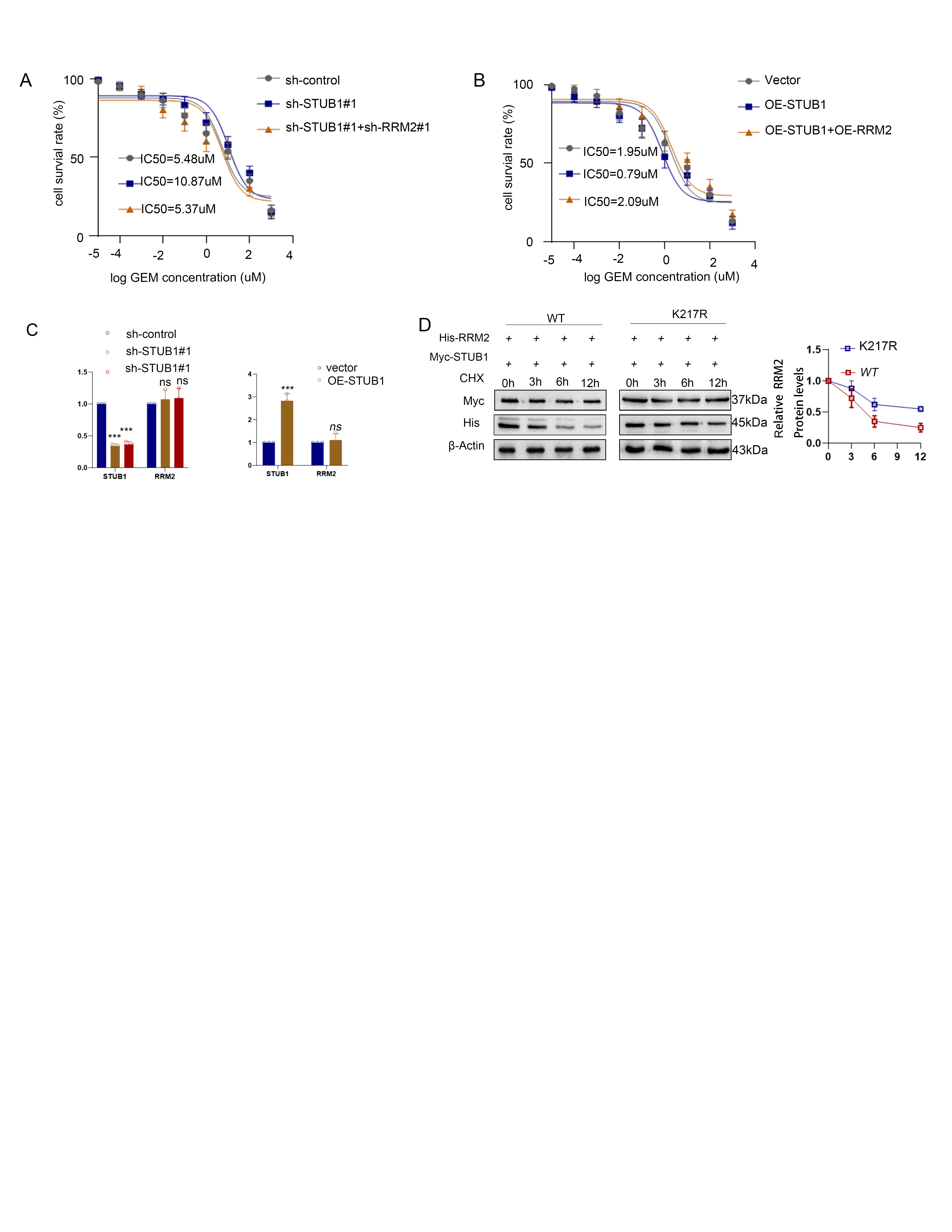


Fig. S4

STUB1 Acts as an E3 Ubiquitin Ligase for RRM2 to Promote Its Degradation **A.** In PANC-1 cells, after knocking down STUB1, RRM2 was further knocked down, and CCK8 assay was used to detect cell sensitivity to gemcitabine. **B.** In SW1990 cells, after overexpressing STUB1, RRM2 was further overexpressed, and CCK8 assay was used to detect cell sensitivity to gemcitabine. **C**. In PANC-1 cells, STUB1 was knocked down, and in SW1990 cells, STUB1 was overexpressed. qPCR was used to detect the expression levels of RRM2. **D.** In HEK293T cells, Myc-STUB1 and His-RRM2 (wild type WT or mutant K217R) were co-transfected, and CHX chase analysis was performed to analyze Myc-RRM2.All in vitro experiments were carried out in three replicates


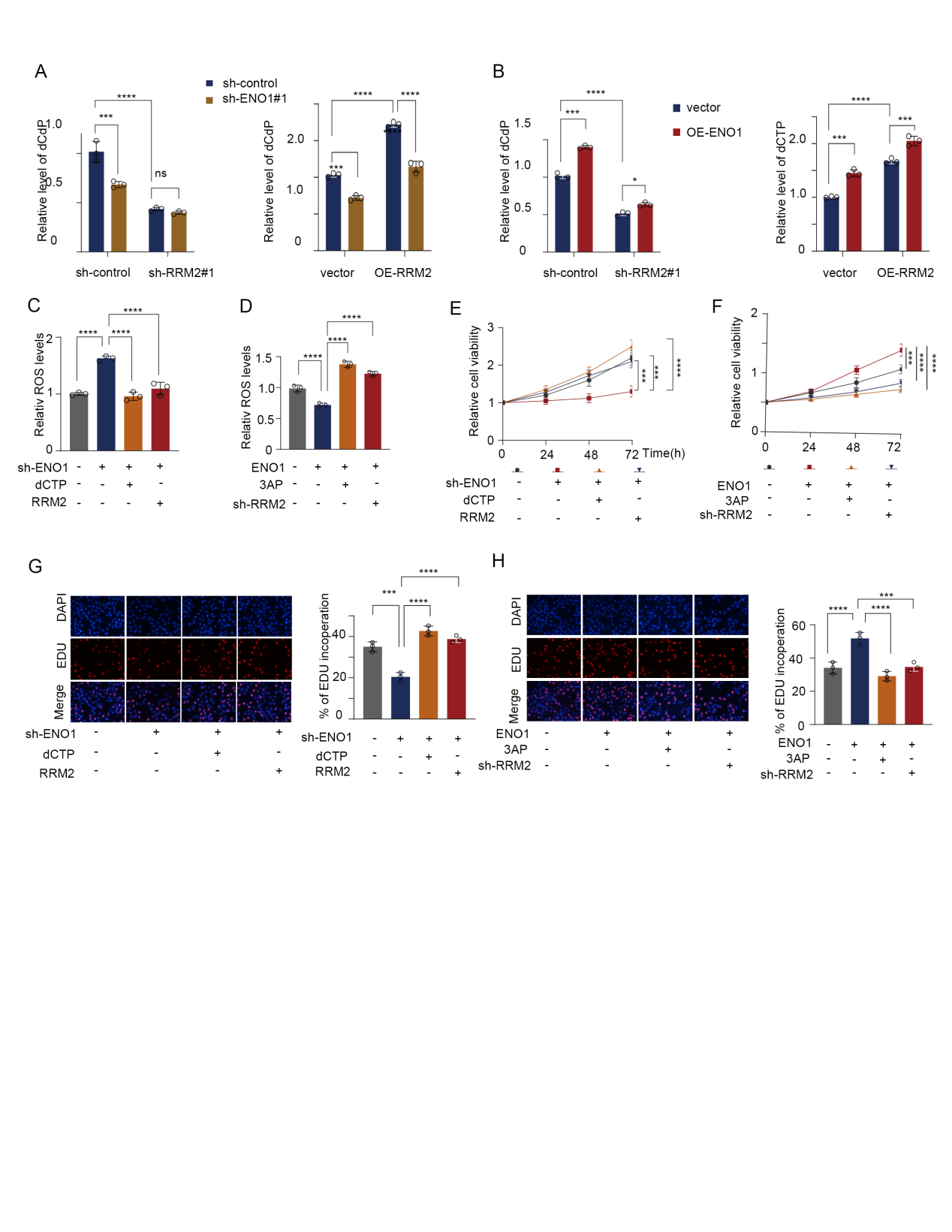


Fig. S5

ENO1-RRM2 Axis Modulate Deoxycytidine Synthesis and Promote Gemcitabine Resistance in Pancreatic Cancer

**A, B**. In PANC-1 cells, ENO1 was knocked down (**A**), and in SW1990 cells, ENO1 was overexpressed (**B**). Subsequently, RRM2 was either knocked down or overexpressed, and dCDP levels were measured. Statistical analysis was performed using a two-tailed Student's t-test.

**C, D**. In PANC-1 cells, after knocking down ENO1 and adding dCTP or overexpressing RRM2, ROS production was measured under gemcitabine induction (5 μM, 24 hours) (**C**). In SW1990 cells, after overexpressing ENO1 and adding RRM2 inhibitor (3AP) or knocking down RRM2, ROS production was measured under gemcitabine induction (2 μM, 24 hours) (**D**). Statistical analysis was performed using a two-tailed Student's t-test. **E, F** In PANC-1 cells, after knocking down ENO1 and adding dCTP or overexpressing RRM2, cell proliferation was assessed using CCK8 under gemcitabine induction (5 μM, 24 hours) (**E**). In SW1990 cells, after overexpressing ENO1 and adding RRM2 inhibitor (3AP) or knocking down RRM2, cell proliferation was assessed using CCK8 under gemcitabine induction (2 μM, 24 hours) (**F**). Statistical analysis was performed using a two-tailed Student's t-test. **G, H.** In PANC-1 cells, after knocking down ENO1 and adding dCTP or overexpressing RRM2, cell proliferation efficiency was assessed using EDU under gemcitabine induction (5 μM, 24 hours) (**G**). In SW1990 cells, after overexpressing ENO1 and adding RRM2 inhibitor (3AP) or knocking down RRM2, cell proliferation efficiency was assessed using EDU under gemcitabine induction (2 μM, 24 hours) (**H**). Statistical analysis was performed using a two-tailed Student's t-test.All in vitro experiments were carried out in three replicates
