# Supplementary material for: ENO1-mediated deoxycytidine synthesis and gemcitabine resistance by stabilizing RRM2 in pancreatic cancer
Source: Cell Death Dis. 2025 Dec 27;17(1):139. doi: 10.1038/s41419-025-08061-6 (PMC12847938; doi:10.1038/s41419-025-08061-6)

fig 1B

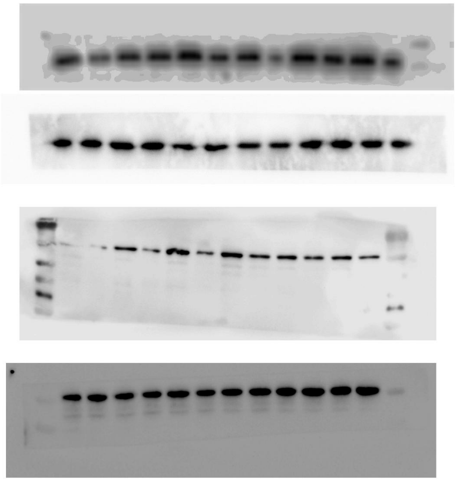

fig 2 B

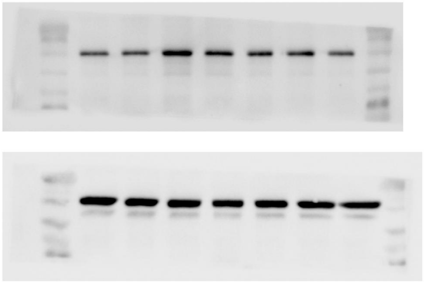

fig 3 B

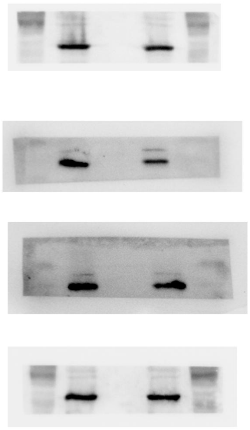

fig 3C

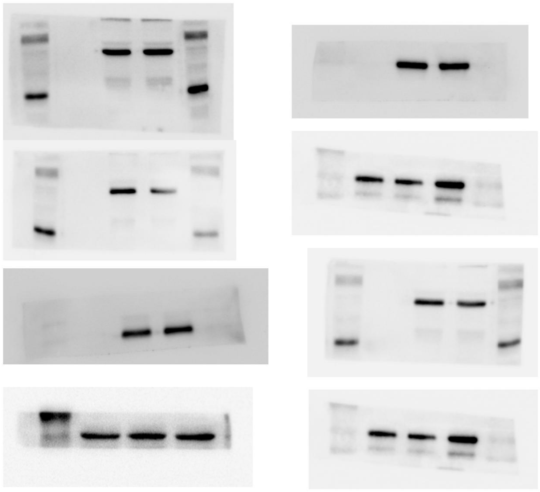

fig3 F

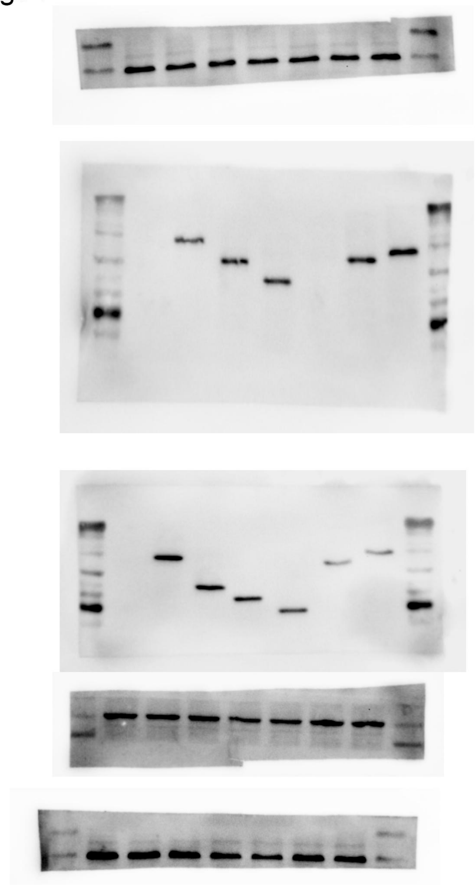

fig 3 G

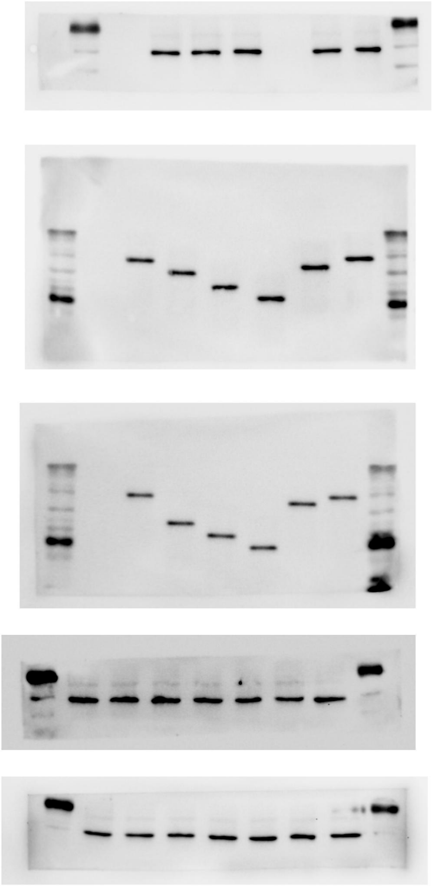

Fig3 H

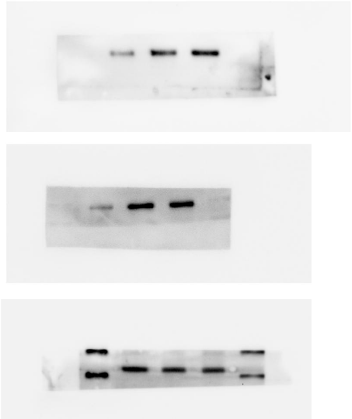

Fig3 K

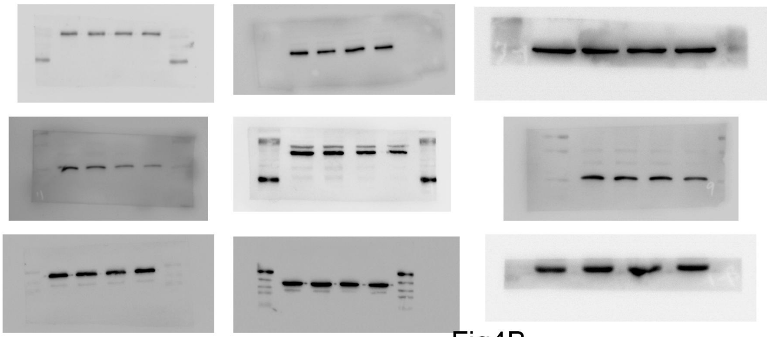

Fig4B

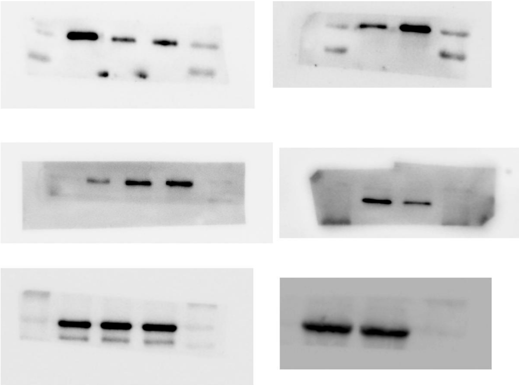

Fig3 L

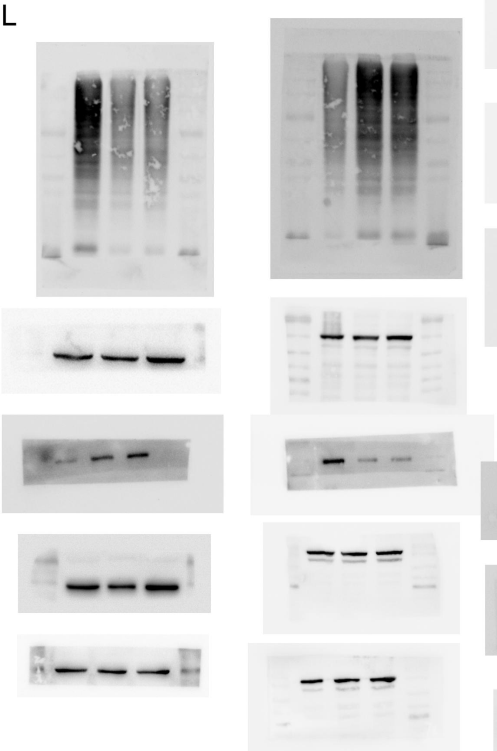

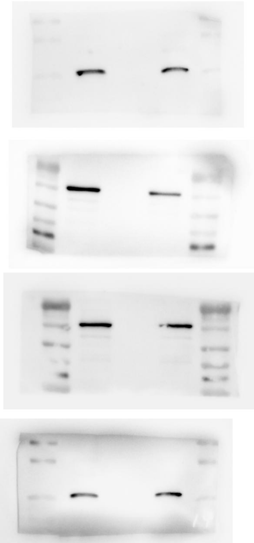

Fig4D

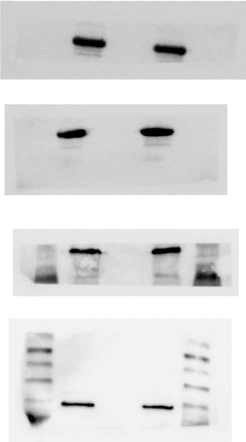

Fig4H

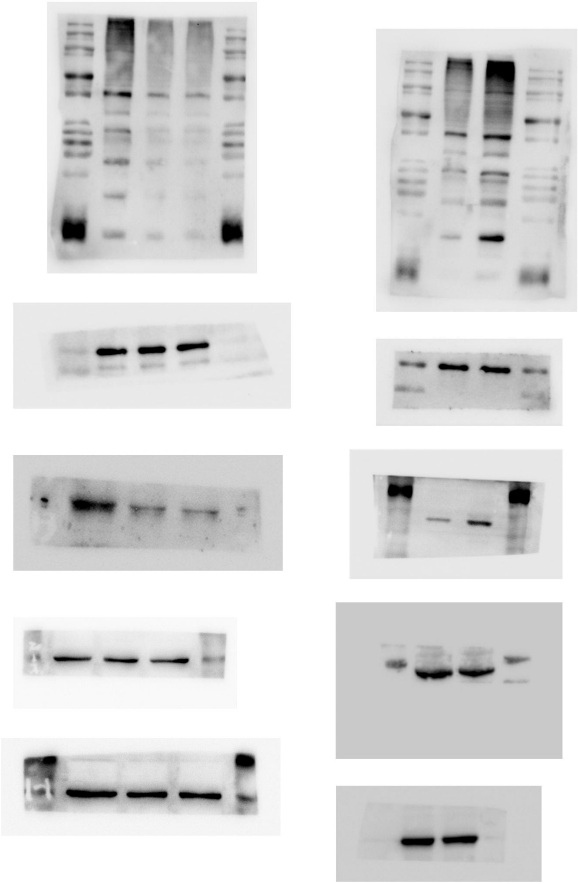

Fig4J

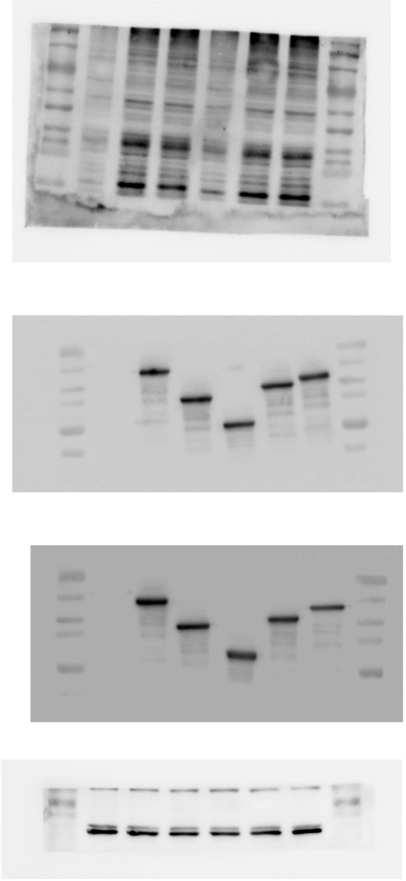

Fig 4 K

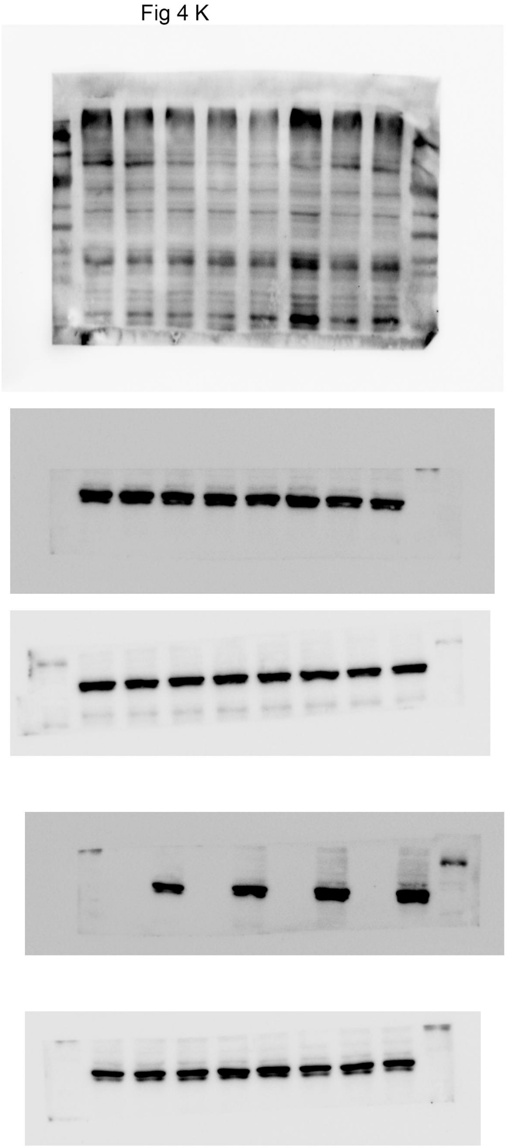

Fig 4 L

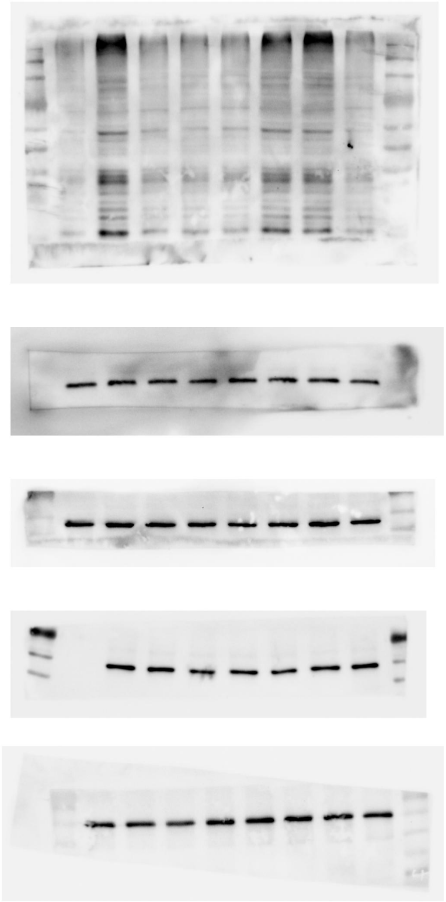

Fig 5A

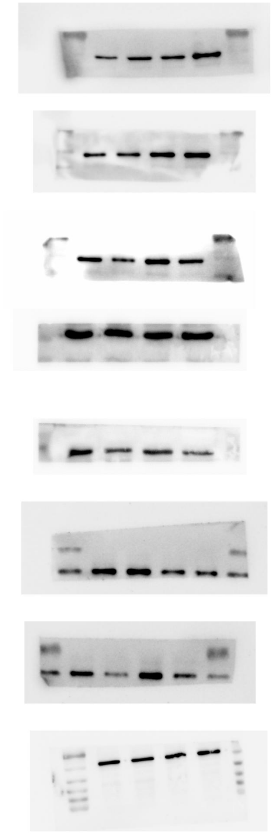

Fig 5B

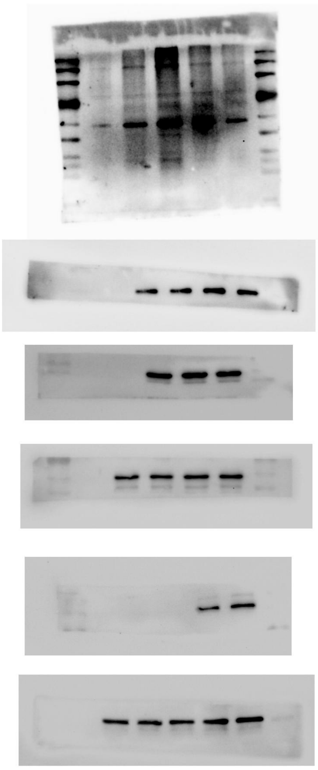

Fig 5C

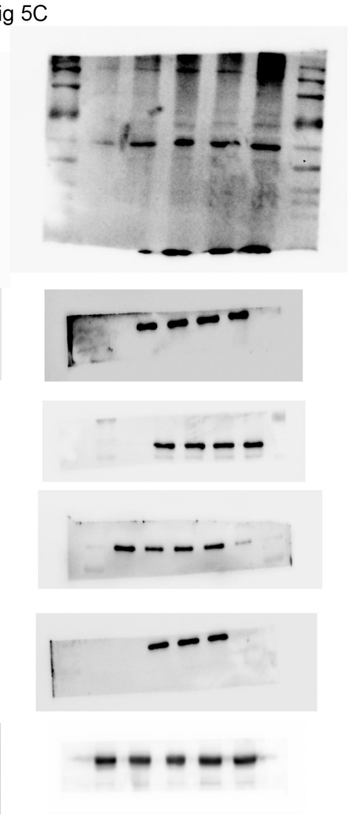

Fig 5D

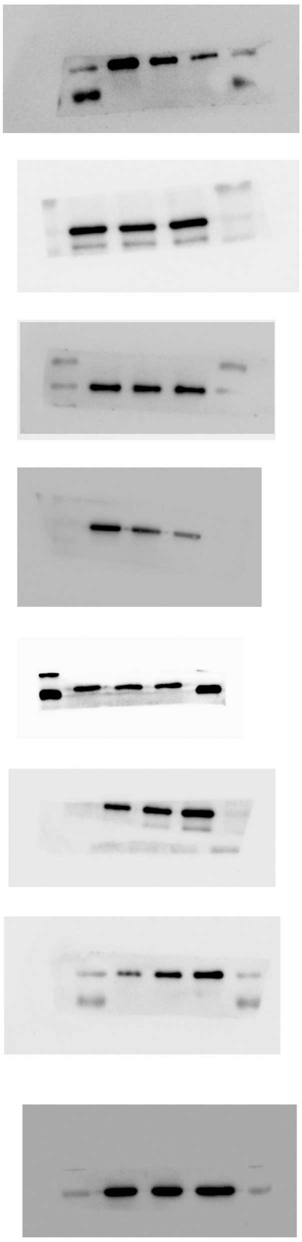

Fig 5E

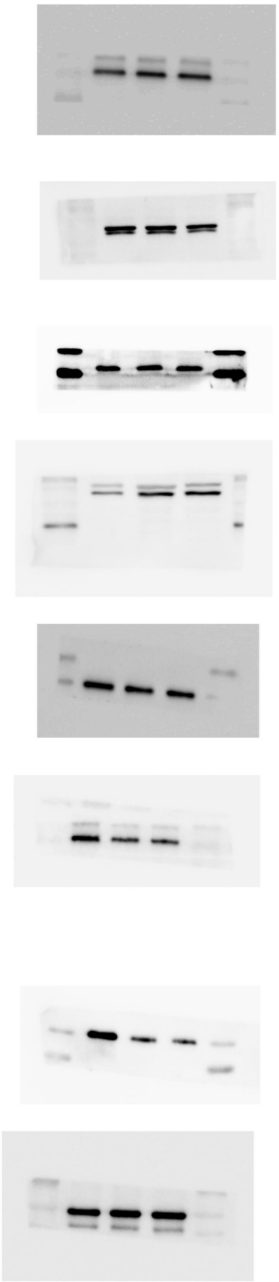

Fig5 F

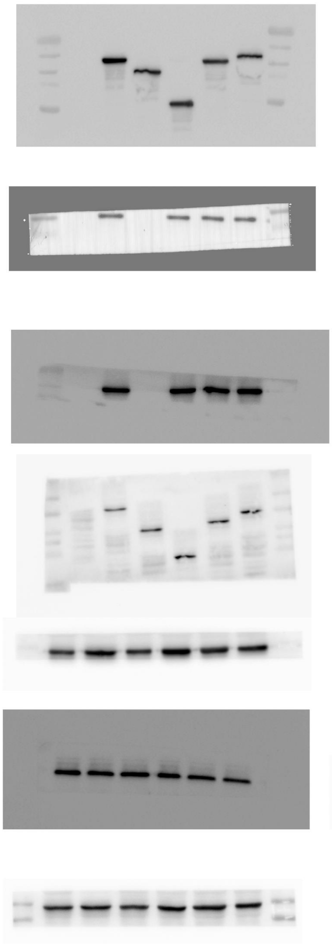

Fig5 G

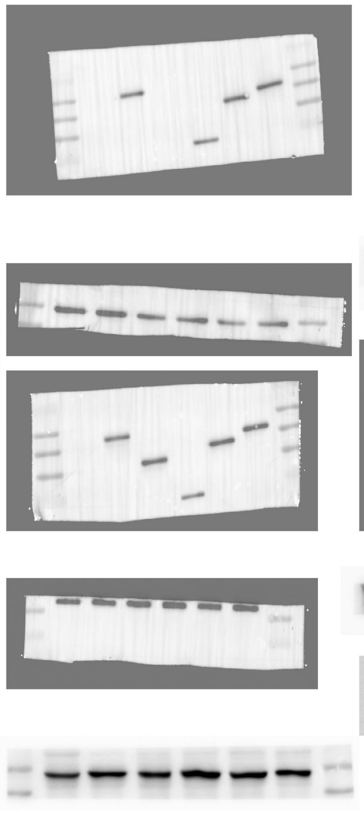

Fig5 H

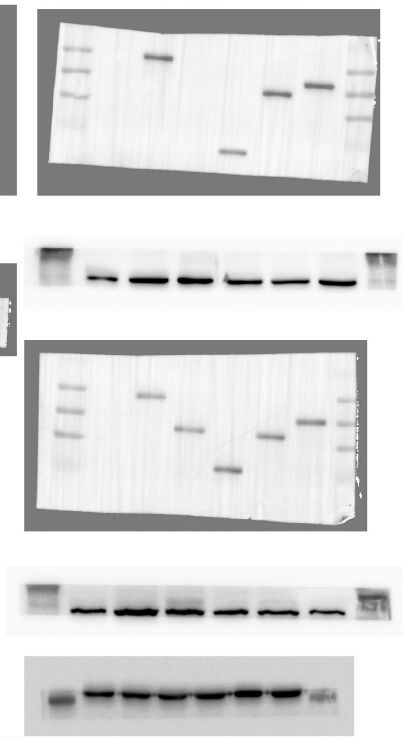

Fig S2 B

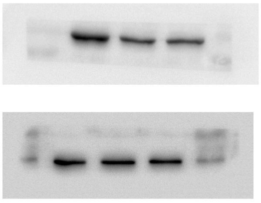

Fig S2 C

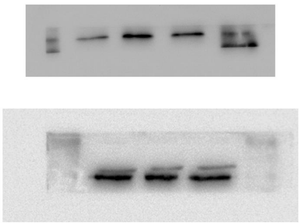

Fig S3 C

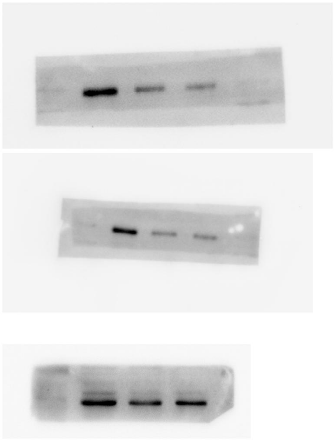

Fig S3 D

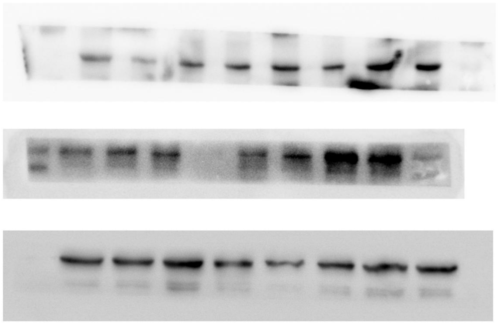

Fig S3 E

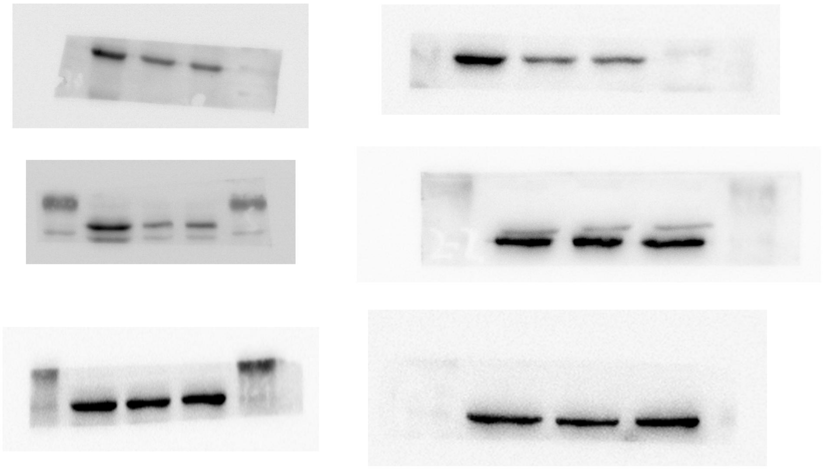

Fig S3E

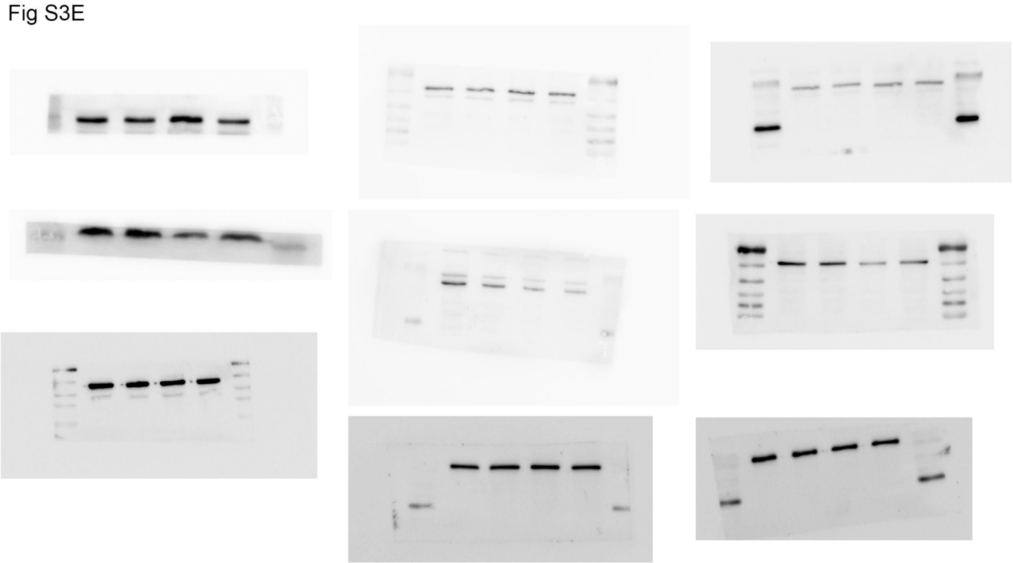

Fig S4 D

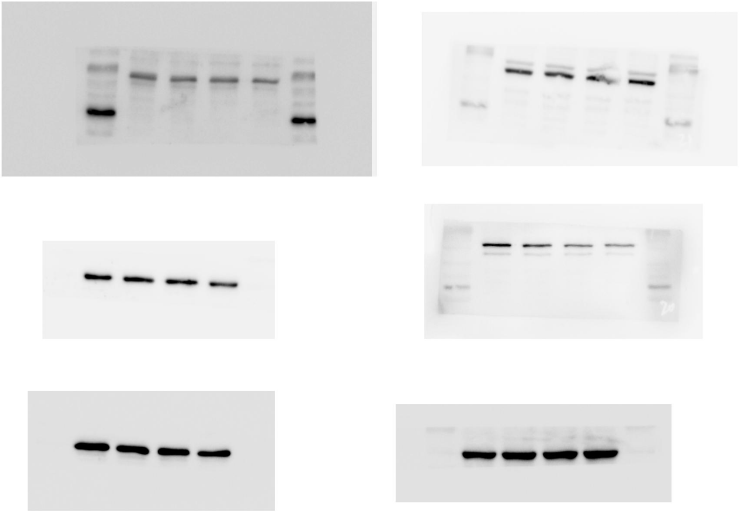

Supplement: Supplementary file 2 — Original Data [file 41419_2025_8061_MOESM2_ESM.pdf]
